# Credit risk prediction model for listed companies based on improved reinforcement learning and Bayesian optimization hyperband

**DOI:** 10.1371/journal.pone.0332150

**Published:** 2025-10-28

**Authors:** Cai Yuanqing, Zhenming Gao, Zhang Jian, Roohallah Alizadehsani, Paweł Pławiak

**Affiliations:** 1 College of Business and Public Management, Wenzhou-Kean University, Wenzhou, China; 2 School of Management, University of Sheffield, Sheffield, United Kingdom; 3 School of Law, The University of Malaya, Kuala Lumpur, Malaysia; 4 Institute for Intelligent Systems Research and Innovation (IISRI) Deakin University, Waurn Ponds, Australia; 5 Department of Computer Science, Faculty of Computer Science and Telecommunications, Cracow University of Technology, Krakow, Poland; 6 Institute of Theoretical and Applied Informatics, Polish Academy of Sciences, Gliwice, Poland; University of Bonab, IRAN, ISLAMIC REPUBLIC OF

## Abstract

The financial sector has experienced swift growth over recent years, leading to the escalating prominence of credit risk among publicly traded companies. Consequently, forecasting credit risk for these firms has emerged as a critical task for banks, regulatory bodies, and investors. Traditional models include the z-score, the logit (logistic regression model), the kernel-based virtual machine (KVM), and neural network approaches. Nevertheless, the outcomes from these methods have often fallen short of expectations. Three major challenges in previous works are feature selection, imbalanced classification, and hyperparameter optimization. This paper presents a method for credit risk prediction for listed companies that uses an off-policy proximal policy optimization (PPO) algorithm for feature selection and imbalanced classification. The off-policy PPO, a reinforcement learning (RL) approach, enhances sample efficiency by more effectively utilizing past experiences during policy updates. This approach improves feature selection and the management of imbalanced classification by optimizing data use, thereby enhancing model training outcomes. Moreover, we use the Bayesian optimization hyperband (BOHB) approach to refine the hyperparameters of the method. BOHB merges Bayesian optimization and Hyperband, significantly speeding up the optimization process. We assess our model using the China Stock Market and Accounting Research (CSMAR), MorningStar, KMV default, Give Me Some Credit (GMSC), and the University of California, Irvine Credit Card Default (UCICCD) datasets. Our experimental findings demonstrate the excellence of the model over existing state-of-the-art models, achieving F-measures of 90.763%, 86.358%, 87.047%, 90.576%, and 89.485% on these datasets. These findings validate the efficiency of the method in economic settings, signifying a major progression in systems for predicting credit risk and enhancing investigative approaches.

## 1. Introduction

As financial markets evolve, the number of publicly traded companies grows, serving as a critical driver of market dynamics. However, alongside the opportunities brought by this expansion, significant risks emerge. Many companies face credit challenges with frequent debt defaults and loan repayment failures. Consequently, accurately forecasting the credit risk associated with listed corporations has become vital. Effective credit risk prediction helps mitigate financial uncertainties and plays a crucial role in stabilizing the broader market. Within financial risk management, assessing corporate credit risk remains a prominent and highly debated topic [1].

Traditional approaches to forecasting credit risk in publicly traded companies include the z-score and logit models [2]. The z-score model employs multivariate statistical techniques, while the logit model incorporates binary explanatory variables (e.g., default versus non-default) to estimate the likelihood of credit risk. This estimation relies on coefficients derived from sample data, with the resulting p-value used to assess the risk level of a company. Among these frameworks, the KMV model stands out as the most widely utilized for credit risk evaluation. It quantifies credit risk through the default distance, enabling the estimation of default probability via mathematical calculations. The KMV model requires large historical datasets of defaults, which can restrict its use [3]. Recently, machine learning (ML), including gradient-boosted decision tree (GBDT) [4], support vector machine (SVM) [5], extreme gradient boosting (XGBoost) [6], and deep learning (DL), such as multi-layer perceptron (MLP) [7], neural network (NN) [8], and long short-term memory (LSTM) [1], have demonstrated effectiveness in managing intricate credit risk prediction scenarios. Credit risks display recurring behaviors and adhere to identifiable patterns, making ML and DL models particularly adept at recognizing the risks. However, ML models struggle with high-dimensional data nonlinear relationships and require extensive feature engineering to perform optimally [9]. DL models automatically detect and utilize complex data patterns by learning hierarchical representations, making them robust and scalable for credit risk prediction without the need for extensive preprocessing. They also excel in handling large volumes of data, adapting more effectively to dynamic market conditions, and improving prediction accuracy over time [10]. Although DL models have outperformed ML methods, these methods still face major challenges, including feature extraction, imbalanced classification, and hyperparameter optimization, which has received little research, especially in risk prediction. This paper aims to present a DL model for credit risk prediction to address these issues.

Feature selection plays a pivotal role in optimizing the performance of DL models, particularly in credit risk prediction [11]. In this field, datasets often comprise many variables, which, without careful management, may conceal rather than highlight critical patterns. Although DL models are adept at automatic feature extraction, this capability does not eliminate the necessity for deliberate feature selection [10]. Relying solely on automatic feature extraction can result in overfitting, as the model may focus on noise rather than crucial patterns, reducing its effectiveness on new datasets. By implementing feature selection strategies, practitioners can minimize the dimensionality of input data, which not only enhances the efficiency of the model but may also reduce training durations [12]. Moreover, prioritizing the most relevant features improves the interpretability of the model, a crucial aspect in financial settings where regulatory compliance and decision-making transparency are imperative. To date, numerous techniques have been employed for feature selection; however, they often struggle with scalability and adaptability in dynamic environments where data distributions evolve [6].

To address the class imbalance issue in earlier models, strategies are implemented in both data and algorithmic groups [13]. At the data level, methods such as enhancing the visibility of underrepresented groups or diminishing the dominance of overrepresented ones are employed to foster a more equitable dataset. Conversely, at the algorithmic level, modifications are introduced to heighten the responsiveness of the model to the minority class. Nevertheless, data-level strategies may lead to overfitting, causing the model to be overly attuned to recognizing frequent or artificial patterns instead of grasping broader, more universal patterns. Additionally, algorithmic modifications to prioritize minority class sensitivity might inadvertently compromise the efficacy of the model in dealing with prevalent cases [14].

Deep RL (DRL) offers advanced approaches for addressing feature selection and class disproportion issues through a flexible reward system [15]. This system prioritizes critical yet often overlooked attributes by rewarding valuable features, thus enhancing the capability of the model to recognize nuanced and vital patterns. Additionally, DRL addresses class imbalance by assigning higher rewards for correctly identifying underrepresented classes, which motivates the model to improve its performance on these minority groups without compromising overall accuracy. This adaptability allows DRL to outperform conventional methods that may not dynamically adjust to evolving data patterns or complex relationships between features [16]. However, DRL models typically face difficulties concerning the off-policy data to increase sample efficiency. Off-policy PPO, effective in gaming, robotics, and control tasks, optimizes policies with data from agent activities to achieve better sample efficiency than on-policy techniques [17]. The adoption of off-policy data helps reduce the costs associated with vast direct engagements, making it highly effective for intricate sequential decision-making works in practical settings. Off-policy strategies save former interactions in a replay buffer, enabling the model to learn from a wide array of previous experiences. This improves the capability and flexibility of the system in dynamic environments and supports the exploration of various approaches [18].

Determining optimal hyperparameters is crucial for enhancing the performance of deep learning models and preventing issues like overfitting or underfitting [19]. Hyperparameters, set before training, significantly influence the prediction ability of a model. However, finding the best hyperparameter settings is challenging, as there is no one-size-fits-all solution for all scenarios. To address this, various optimization methods have been developed, including grid search [20], Bayesian optimization [21], evolutionary algorithms [22], and hyperband [23]. Grid search exhaustively evaluates all possible combinations within a predefined range of values, thoroughly covering the parameter space. However, this method consumes substantial resources and becomes inefficient for models with a large number of hyperparameters. In contrast, Bayesian optimization views the parameter space through a probabilistic lens, using past results to strategically choose the next points for evaluation. Although more efficient than grid and random searches, it can still be limited by local optima and heavily relies on well-selected prior distributions [24]. Evolutionary algorithms mimic natural selection among sets of hyperparameters, effectively exploring complex spaces but at the expense of speed and computational resources. Hyperband efficiently manages resources by quickly identifying and focusing on promising hyperparameter configurations, discarding fewer effective ones. BOHB combines the predictive power of Bayesian optimization with the resource-efficient strategy of Hyperband, thereby overcoming the limitations of traditional methods, such as the intensive computational demands of grid searches and the challenges of local optima in Bayesian optimization. By efficiently pruning less favorable options and focusing on areas with potential, BOHB offers a sophisticated and effective approach to hyperparameter tuning, particularly suited for complex models, such as those used in credit risk prediction [25].

This paper presents a groundbreaking model for credit risk prediction in listed companies, integrating an off-policy PPO strategy for feature selection and class imbalance management, as well as a BOHB method for optimizing hyperparameters. The model utilizes MLP as its policy network, trained with off-policy PPO using a specially designed reward function to highlight key features and address class imbalances. The experimental results demonstrate that the proposed method enhances prediction accuracy. The contribution points of this paper are as follows:

- This model employs an off-policy PPO approach specifically designed for feature selection. Using a tailored reward function within the off-policy PPO framework, the model strategically emphasizes identifying and selecting crucial features within the dataset. This methodology highlights significant predictors and streamlines the model by focusing on relevant data, potentially enhancing the efficiency and accuracy of credit risk prediction.
- Another significant contribution of the model is the application of off-policy PPO to address the challenge of class imbalance, a common issue in credit risk datasets where some classes are underrepresented. The tailored reward function within the off-policy PPO is designed to provide higher rewards for correctly predicting instances of the minority class, thereby motivating the model to improve its sensitivity and accuracy towards these less-represented classes without compromising overall performance.
- The model utilizes the BOHB method to efficiently optimize its hyperparameters. BOHB combines the strengths of Bayesian optimization and the Hyperband technique, allowing for a more strategic exploration and exploitation of the hyperparameter space. This approach accelerates the optimization process while ensuring that the model operates with the most effective set of parameters, thereby enhancing the performance of the model in predicting credit risk with greater accuracy and reliability.

The article is organized as follows: Section 2 provides a review of pertinent studies related to predicting credit risk, while Section 3 describes the proposed methodology. Section 4 showcases the results and analysis of experiments conducted, and Section 5 offers a conclusion to the article.

## 2. Related work

In the evolving landscape of financial analytics, credit risk prediction remains a pivotal challenge that has garnered considerable attention due to its significant implications for banks and investors. The related work in this domain encompasses various methodologies that have evolved progressively from traditional machine learning techniques to sophisticated deep learning models, reflecting a broader shift toward more complex, data-driven approaches. Section 2–1 outlines the foundational machine learning methods that have historically been employed to predict credit risk, setting the stage for the introduction of deep learning models in Section 2–2, which harness enhanced computational power and larger datasets to improve prediction accuracy. Finally, Section 2–3 critically analyzes how these existing models compare to our proposed model, highlighting key differences and our unique contributions that address specific gaps in the current literature.

### 2.1 Machine learning

Machine learning uses techniques like extreme gradient boosting (XGBoost), logistic regression (LR), and random forest (RF) to improve the accuracy and personalization of drug recommendations. In 2023, Kanaparthi [11] introduced an innovative method for assessing credit risk that utilizes ensemble machine learning techniques to support financial institutions. Their approach uses the information gain method to preprocess data and select significant features. Notably, the first ten features train various gradient boosting models, including XGBoost, RF, and categorical boosting (CatBoost). This method is benchmarked against algorithms such as AdaBoost and Random Forest, underscoring its utility in effectively evaluating borrower reliability. Wang et al. [26] addressed the challenge of predicting credit risk for small and medium-sized enterprises (SMEs) in supply chain finance (SCF) by developing forecast models that utilize an imbalanced sampling strategy and machine learning techniques. Their approach involves the fusion of multi-source information, integrating financial, operational, innovation, and negative event data as predictors. To enhance the accuracy of predictions, they employ cost-sensitive learning extensions within a random forest framework. Rao et al. [6] addressed credit risk assessment for personal auto loans on online peer-to-peer (P2P) platforms with a machine learning-based method. They used the smote-tomek link algorithm to balance the dataset and then applied an advanced filter-wrapper feature selection method to pinpoint critical assessment indices. The study further develops a particle swarm optimization- XGBoost (PSO-XGBoost) model to evaluate credit risk more effectively than traditional models like RF and LR.

In 2024, Pang et al. [27] proposed a three-way decision method for credit risk prediction, integrating prospect theory and evidence theory. They introduce a sample-weighted support vector data description (SW-SVDD) model to address classification challenges and predict default statuses. The method classifies samples into definite and uncertain boundaries, using SW-SVDD for clear cases and a combined decision approach for uncertain cases, enhancing accuracy and interpretability. Yu et al. [28] proposed a bank credit risk prediction approach that leverages dimensionality reduction techniques, including principal component analysis (PCA) and t-distributed stochastic neighbor embedding (T-SNE), alongside distributed models such as LightGBM and XGBoost, as well as deep learning models like TabNet. The method integrates synthetic minority oversampling edited nearest neighbor (SMOTEENN) to balance datasets, enabling precise identification of high-quality credit card applicants and optimizing the efficiency of credit risk evaluation processes. Su [29] employed the random forest algorithm for credit risk modeling and prediction, leveraging its ensemble capabilities to handle large-scale credit-related data. They preprocess the data using feature engineering, which includes handling missing values, feature selection, and standardization. The model is optimized through multiple decision trees, effectively identifying potential default risks in financial technology applications. Hua et al. [30] presented a federated three-way decision incremental naive bayes (FTwNB) model for credit risk assessment in banking. This approach integrates federated learning to eliminate data barriers, asymmetric encryption for data security, and a three-way classification method to better handle uncertain cases. Incremental learning further refines model performance by filtering low-quality training samples.

In 2025, Hou et al. [31] developed a credit risk prediction model for SMEs in SCF using a light gradient boosting machine (LightGBM), optimized through an improved sparrow search algorithm (ISSA) that integrates fractional calculus and Cauchy–gaussian mutation. Yu et al. [32] introduced a hybrid method called two-stage spectral clustering and SVM (TSC-SVM), which combines spectral clustering and semi-supervised SVM with multi-view unsupervised learning to enhance balance and robustness in credit risk assessment. Zhang [33] presented a framework that applies the SMOTE method to address the class imbalance and then compares LR, RF, and SVM to determine the most suitable prediction model. Li et al. [34] proposed an enhanced SVM framework using adaptive Lq-norm SVM (Lq-SVM), Gaussian kernels, and evolution strategy (ES) to optimize hyperparameters for small and nonlinear credit datasets. Alamsyah et al. [35] designed a credit scoring model using social media analytics, leveraging demographic, psycholinguistic, and personality features extracted from LinkedIn profiles to build machine-learning models for evaluating creditworthiness in the absence of traditional financial data. Gasmi et al. [36] introduced a feature selection-based classification model for credit risk, employing five selection algorithms, including speed-constrained multi-objective particle swarm optimization (SMPSO) and non-dominated sorting genetic algorithm II (NSGA-II), and evaluating the selected features through classifiers such as k-nearest neighbors (KNN), SVM, and artificial neural network (ANN).

### 2.2 Deep learning

In 2023, Yang et al. [37] developed an enhanced deep neural network (HDNN) algorithm tailored for high-dimensional corporate credit risk prediction. Addressing the common issue of redundant and irrelevant data, their method incorporates both lasso regularization (L1) and ridge regularization (L2) to refine feature selection and improve model accuracy. The innovation of their approach lies in proving theoretically that L1 regularization does not affect batch normalization layers and in demonstrating how adding L2 constraints can compensate for this. Li et al. [1] proposed a credit risk prediction model for listed companies that integrates convolutional neural network-LSTM (CNN-LSTM) with an attention mechanism. By combining a CNN for feature extraction and an LSTM for time-series prediction, the model addresses data complexity and improves computational efficiency. The attention mechanism assigns weights to optimize predictions, enhancing accuracy and providing meaningful insights for credit risk assessment. Cong [38] presented a bank credit risk prediction model integrating CNN and XGBoost with multi-criteria decision-making (MCDM) techniques. The CNN identifies critical features, while XGBoost enhances prediction accuracy. MCDM establishes a comprehensive evaluation framework for multi-criteria analysis, providing practical solutions for credit risk assessment.

In 2024, Zhang et al. [39] introduced an enterprise credit risk prediction method using an attention-based CNN-BiLSTM hybrid neural network. The model incorporates logistic regression outputs and constructs a prediction index system based on profitability, debt-paying ability, growth ability, operational efficiency, and basic enterprise information. This approach leverages historical financial data to enhance accuracy and support effective risk management for listed real estate enterprises. Shi et al. [40] proposed a hybrid KNN-graph neural network (kNN–GNN) model for credit risk prediction, integrating graph representation learning to enhance performance using only internal information. The method involves constructing edges through kNN and implementing GNN for node classification to distinguish risk cases. The approach combines unsupervised graph transformation with supervised classification, offering a novel solution for improving predictive accuracy in credit risk analysis. Meng et al. [41] presented an unthresholded recurrence plot-CNN (URP-CNN) model for bond credit risk evaluation of Chinese listed companies, combining URP for spatial association extraction and CNN for feature classification. This hybrid approach leverages the representation capabilities of URP and the classification strengths of CNN to effectively address the complexity of bond default prediction, ensuring robust performance. Yang et al. [42] proposed a federated transfer learning approach with frozen network parameters to enhance model convergence and accuracy. By freezing two to four network layers, this method accelerates parameter sharing securely through homomorphic encryption. It effectively balances computational efficiency and model precision, enabling rapid convergence in federated environments while maintaining data confidentiality for credit risk prediction. Berhane et al. [43] proposed a hybrid machine learning model combining CNN with logistic regression, gradient-boosting decision trees, and KNN for credit risk prediction in peer-to-peer lending. Addressing data imbalance through synthetic oversampling, the model replaces the fully connected layer of CNN with these algorithms, enhancing classification performance and supporting effective credit risk appraisal in P2P systems. Xia et al. [44] developed a deep-learning model integrating CNNs with LSTM networks to predict default risk in peer-to-peer microlending platforms. The method captures spatial and temporal patterns in lending data, employing a hybrid feature selection approach and ensemble learning strategy with gradient boosting and random forest classifiers to enhance predictive accuracy and transparency. Furthermore, Shapley additive explanations (SHAP) and local interpretable model-agnostic explanations (LIME) methods enhance the interpretability of the model, providing insights into the key factors that affect credit risk predictions. Pavitha and Sugave [45] introduced an explainable multistage ensemble one-dimensional (1D) CNN for credit risk prediction, integrating a multistage approach with convolutional architecture to enhance performance and interpretability. The model utilizes explainability techniques to provide transparent lending decisions, enabling financial institutions to assess risk more effectively, reduce defaults, and ensure a stable and inclusive financial ecosystem while maintaining high predictive accuracy. Wang et al. [46] proposed a multistage approach to enhance credit risk prediction using a ResNet-LSTM hybrid neural network. The method involves balancing class distribution with the k-means-SMOTE algorithm, extracting features with ResNet, and leveraging a two-layer LSTM for deep information mining. Optimization is achieved through the IDWPSO algorithm, improving model classification performance for imbalanced datasets. Xiong et al. [47] proposed a dynamic credit risk assessment model utilizing a deep Q-network (DQN) framework, thereby addressing the limitations of static models. Incorporating prioritized experience replay and ring noise, the model captures nonlinearities and correlations in credit data, enabling it to adapt to economic shifts. This approach enhances credit risk management, supporting inclusive lending while mitigating default risks in dynamic financial environments. Jiang et al. [48] proposed a credit risk prediction strategy that utilizes generative adversarial networks (GANs) to generate synthetic samples of rare financial risk events. The model addresses data imbalance by adding synthetic samples of rare events to the training dataset.

In 2025, Hartomo et al. [49] proposed a credit risk assessment framework that combines the tabular transformer (TabTransformer) with a weighted loss function to address the class imbalance. SHAP was integrated to enhance interpretability, facilitating the creation of fair, transparent, and balanced credit predictions using deep learning models for tabular data. Ye et al. [50] designed a scorecard system combining the TabTransformer model and an improved LIME method to enhance prediction and interpretability. This approach captures nonlinear relationships and provides localized explanations for better credit decision-making. Adiputra et al. [51] applied GAN-based oversampling methods, conditional tabular GAN (CTGAN), copula GAN, wasserstein GAN with gradient penalty (WGAN-GP), and data regularized GAN (DraGAN), to mitigate class imbalance in multi-class credit scoring by generating synthetic samples for minority categories. These samples were evaluated using both classical and ensemble machine-learning models. Wang et al. [52] proposed a hybrid model that integrates bidirectional encoder representations from transformers (BERT) to extract features from legal judgment texts and employs an auxiliary classifier, wasserstein GAN with gradient penalty and spectral attention (ACWGAN-GPSA), to address data imbalance. This method combines textual, financial, and corporate information to improve credit risk prediction in SCF. Han et al. [53] introduced a deep learning model for credit risk that uses adaptive temporal fusion, a heterogeneous graph neural network (GNN), and an attention-based output layer to ensure symmetry in financial data modeling across time and structure. Shen et al. [54] proposed a hybrid architecture that combines temporal convolutional networks (TCNs) with a dilated transformer to better capture both long-range dependencies and local features in high-dimensional financial data. Zhang et al. [55] developed a domain-adaptation-based multistage ensemble learning (DAMEL) method for assessing the credit risk of SMEs in SCF, addressing challenges such as limited data, redundant features, and domain shifts using techniques like bagging, random subspace selection, domain adaptation, and dynamic model selection.

### 2.3 Differences compared to the proposed model

Table 1 presents a detailed review of the most sophisticated machine learning and deep learning models for credit risk prediction. The main challenges in using traditional machine learning methods for credit risk prediction include limited feature learning capabilities, which hinder the ability of the model to autonomously extract and interpret complex patterns and interactions in the data. Traditional algorithms often require extensive manual feature engineering and selection, which can be time-consuming and may not capture all the predictive subtleties. They also struggle with scalability and generalization when applied to large or diverse datasets, making them less effective as data volume and variety grow. Additionally, these models can underperform in non-linear problem spaces where relationships between variables are not straightforward or are hidden. Deep learning models, on the other hand, excel in these areas by leveraging layers of learning that progressively extract higher-level features from raw input automatically. This enables them to handle vast amounts of data with intricate structures, learn non-linear relationships, and improve prediction accuracy and reliability in credit risk assessments.

**Table 1:** Comparison of machine learning and deep learning models for credit risk prediction.

Study	Method	Key focus	Limitation
Kanaparthi [11]	Ensemble methods with XGBoost, RF, and CatBoost	Enables credit risk prediction in underbanked populations	Requires significant data preprocessing and feature selection
Wang et al. [26]	Multi-source info fusion with CSL-RF for the credit risk of SMEs	Integrates cost-sensitive learning with random forest	May not address all types of credit risks of SMEs
Rao et al. [6]	PSO-XGBoost with Smote-tomek and filter-wrapper	Integrates PSO for feature selection and model optimization	May not generalize across different datasets
Pang et al. [27]	Three-way decision using prospect and evidence theory	Enhances interpretability in uncertain credit risk boundaries	Limited exploration of broader real-world applications
Yu et al. [28]	PCA-SMOTEENN with LightGBM for credit risk prediction	Combines dimensionality reduction and resampling for balanced predictions	Limited focus on non-credit card financial products
Su [29]	RF with feature engineering for credit risk	Applies preprocessing and feature selection for robust predictions	Limited to structured credit-related data
Hua et al. [30]	FTwNB	Integrates federated learning and three-way decisions for secure credit classification	Limited to scenarios requiring federated data sharing
Hou et al. [31]	LightGBM with ISSA	Uses ISSA to tune LightGBM for improved global optimization	Depends on handcrafted ISSA design and parameter sensitivity
Yu et al. [32]	TSC-SVM with spectral clustering and semi-supervised SVM	Combines spectral clustering with SVM to handle imbalanced data	Relies on parameter tuning and initial cluster quality
Zhang [33]	SMOTE with LR, RF, and SVM	Uses SMOTE to address class imbalance before classification	Depends on AUC for model selection without deeper interpretability
Li et al. [34]	Adaptive Lq-SVM with Gaussian kernel and ES	Integrates SVM tuning, kernel selection, and Lq loss function	Needs extensive tuning and multiple components to configure
Alamsyah et al. [35]	ML with social media-based feature extraction	Enables credit assessment for individuals without a credit history	Depends heavily on the quality and availability of social data
Gasmi et al. [36]	Multi-algorithm feature selection with classifiers	Identifies minimal predictor sets for better model performance	Effectiveness depends on data quality and feature relevance
Yang et al. [37]	HDNN algorithm for high-dimensional credit risk	Solves L1 regularization issue in batch normalization	May introduce bias with L2 constraints on L1 regularization
Li et al. [1]	CNN-LSTM with attention mechanism for credit risk	Combines time-series analysis and attention for weighted predictions	Relies heavily on high-quality historical data
Cong [38]	CNN-XGBoost with MCDM for bank credit risk	Combines neural networks with multi-criteria evaluation for robust	Limited exploration of cross-border banking systems
Zhang et al. [39]	Attention-based CNN-BiLSTM with logistic regression features	Combines deep learning and logistic insights for risk prediction	Focused on listed real estate enterprise data only
Shi et al. [40]	kNN-GNN hybrid for credit risk prediction	Integrates graph representation for unsupervised-supervised classification framework	Limited to datasets with well-defined internal features
Meng et al. [41]	URP-CNN for bond credit risk evaluation	Integrates recurrence plots and CNN for spatial data analysis	Limited application to non-bond financial datasets
Yang et al. [42]	Federated transfer learning with frozen network layers	Enhances convergence via parameter freezing and secure transfer	Limited to specific federated learning scenarios
Berhane et al. [43]	Hybrid CNN with LR, GBDT, and k-NN for P2P lending	Combines CNN with traditional models for risk assessment in P2P lending	Limited generalizability to non-P2P lending datasets
Xia et al. [44]	CNN-LSTM with hybrid feature selection for P2P microlending	Integrates spatial-temporal patterns and interprets key risk factors using SHAP and LIME	Limited to data-rich P2P lending platforms
Pavitha and Sugave [45]	Multistage ensemble with 1D CNN for credit risk	Balances prediction accuracy with transparent, interpretable decision-making	Limited to datasets from Indian financial institutions
Wang et al. [46]	ResNet-LSTM with K-Means-SMOTE and IDWPSO optimization	Combines feature extraction, balancing, and optimization for credit risk prediction	Focused on specific imbalance ratios in datasets
Xiong et al. [47]	Deep Q-network with prioritized replay for credit risk	Captures non-linearities and adapts to dynamic financial scenarios	Challenges in interpretability and potential bias
Jiang et al. [48]	GAN-based synthetic data generation	Enhances rare event prediction in financial risk monitoring	GAN training may introduce unrealistic or noisy synthetic data
Hartomo et al. [49]	TabTransformer with weighted loss	Enhances fairness and interpretability in credit prediction	May require extensive tuning for different datasets
Ye et al. [50]	TabTransformer with enhanced LIME	Improves interpretability and captures nonlinear feature effects	May rely on labeled structured data
Adiputra et al. [51]	GAN-based oversampling with ML	Enhances minority class representation using synthetic data	May generate noisy or unrealistic samples in rare categories
Wang et al. [52]	BERT with ACWGAN-GPSA	Merges textual and structured data for risk prediction	GAN component may produce low-quality or biased synthetic samples
Han et al. [53]	Temporal GNN	Integrates time fusion, graph learning, and interpretable attention	Requires extensive training data and may face overfitting risks
Shen et al. [54]	TCN with a dilated transformer	Captures long-term trends and local features in financial sequences	May require high computational resources during training
Zhang et al. [55]	Domain-adaptation-based multistage ensemble learning	Handles scarcity, redundancy, and distribution shifts in SME credit data	Performance may vary across datasets with different domain characteristics

Deep learning techniques are promising but still require improvements in efficiency and utility. Traditional methods often lack effective feature selection, including irrelevant or redundant features that obscure key patterns and reduce model accuracy. Furthermore, the prevalent class imbalance in datasets related to Credit risk usually leads to models that favor the majority class, thus neglecting the less represented but critical minority classes. Additionally, optimizing hyperparameters remains a substantial challenge; traditional techniques often involve manual adjustments that are prone to inaccuracies and inefficiencies.

To address the challenges of deep learning models in credit risk prediction, this article presents a novel approach that leverages off-policy PPO and the BOHB method to enhance feature selection, imbalanced classification, and hyperparameter tuning. The off-policy PPO approach leads to improved feature selection and better management of imbalanced classification by optimizing data use, thereby enhancing model training outcomes. Moreover, BOHB combines Bayesian optimization and Hyperband, significantly speeding up the optimization process.

## 3. Methods and material

This section discusses developing a model for credit risk prediction for listed companies through an MLP. It highlights an advanced approach to increase prediction precision by incorporating a feature selection mechanism that leverages off-policy PPO and optimizing hyperparameters via the BOHB method. Off-policy PPO is advantageous as it allows the model to learn from many past experiences, not just recent interactions. This improves the speed and efficiency of the learning process. This is particularly useful in credit risk, where data may not always be balanced. Off-policy PPO also provides the flexibility to adapt to complex and dynamic risk behavior patterns. The BOHB algorithm provides a robust and efficient approach for tuning hyperparameters in complex settings, such as off-policy PPO, by quickly eliminating less effective options and focusing on more promising ones.

**Fig 1:**
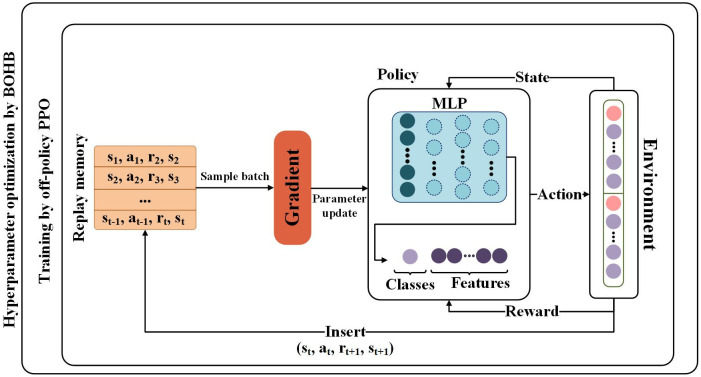
Schematic of the proposed credit risk prediction model employing off-policy PPO for feature selection and imbalanced classification and the BOHB method for optimal hyperparameter tuning.

Fig 1 depicts the detailed architecture of the model used for credit risk prediction. In this model, an input consisting of n features is processed by the MLP network. This network is configured to produce c+n outputs, where c=2 represents the categories (risk and non-risk) and n shows the number of input features.

### 3.1 Feature selection and prediction

Let $Data=\{(x_{i},y_{i}{\}}_{t=\text{1}}^{T}$ be the input dataset, where $x_{i}\in R^{n}$ is an input instance composed of n features $F\;=\;\{f_{\text{1}},\;...,\;f_{n}\}$, and $y_{i}\in \{\text{0},\text{1}\}$ is the binary outcome (e.g., non-default or default). T shows the overall number of samples in Data. We define the environment of the RL agent used for feature selection and classification as follows:

- State space (S): Each state $s_{t}\in S$ is represented as a tuple ($x_{t},y_{t},F_{t}$), where $F_{t}\subseteq F$ is the subset of features selected by the agent up to time step t.
- Action space (A): The action set is defined as $A=A_{f}\cup A_{c}$, where $A_{f}=\left\{f_{i}\right|\;f_{i}\in F\backslash F_{t}\}$ includes feature selection actions that allow the agent to add a new feature to the selected set. $A_{c}=\{a_{\text{0}},a_{\text{1}}\}$ includes classification actions where the agent predicts class 0 or class 1.
- Reward function (r): The reward function r: S×A→R is designed to guide the agent towards cost-effective feature selection and accurate classification, particularly emphasizing sensitivity to minority classes:

$$r\left(s_{t},a_{t}\right)=\;\left\{ \begin{array}{c} -\lambda \times c\left(f_{i}\right)\;\;\;\;\;\;if\;a_{t}\in A_{f},\;a_{t}=f_{i}\\ +\text{1}\;\;\;\;\;\;\;\;\;\;\;\;\;\;\;\;\;\;\;if\;a_{t}\in A_{c},\;s_{t}\in D_{O},a_{t}=y_{t}\\ -\text{1}\;\;\;\;\;\;\;\;\;\;\;\;\;\;\;if\;a_{t}\in A_{c},\;s_{t}\in D_{O},\;a_{t}\neq y_{t}\\ +\gamma \;\;\;\;\;\;\;\;\;\;\;\;\;\;\;\;\;\;\;if\;a_{t}\in A_{c},\;s_{t}\in D_{N},\;a_{t}=y_{t}\\ -\gamma \;\;\;\;\;\;\;\;\;\;\;\;\;\;\;if\;a_{t}\in A_{c},\;s_{t}\in D_{N},\;a_{t}\neq y_{t} \end{array}\right.\;$$ (1)

where λ∈[0,1] is a cost-balancing parameter, and $c(f_{i}\backslash )$ is the cost of selecting i-th feature $f_{i}$ from F. $D_{O}$ and $D_{N}$ denote minority and majority class samples respectively. γ<1 acts as a weighting factor that intensifies both the incentive and penalty linked to decisions involving the majority class. The reward mechanism assigns higher values to correct predictions for the minority class than for the majority class. More precisely, the agent receives a reward of +γ for a correct classification of a majority instance, while a correct decision for a minority instance results in a reward of only +1. Conversely, misclassifications result in penalties of −γ for the majority class and −1 for the minority class. This reward strategy increases the attention of the model to the minority class and lowers bias toward the majority. Throughout training, the reinforcement learning agent continuously adjusts the policy in response to these feedback signals. It learns to detect useful and detailed patterns in the minority class by following the custom reward signals. This iterative learning dynamic is essential for building a model that not only performs well overall but also maintains accuracy across both dominant and infrequent classes.

This reward function is designed to be general rather than limited to a specific domain. The reward function includes three main parts: a penalty for selecting costly features, a reward rule that depends on class labels, and adjustable parameters. The feature selection penalty, defined as$\;-\lambda \times c\left(f_{i}\right)$, indicates how the method considers feature costs and can be applied in any setting where resources are limited. The reward rule gives different values to the two classes. When γ∈(0,1), it increases the effect of minority class predictions. This allows the model to learn from underrepresented examples while still considering the majority class. Although λ and γ can be adjusted for each domain, the overall structure of the reward function is general. As such, this reward framework can be applied to other reinforcement learning classification tasks that involve feature selection, cost, and class imbalance [56,57].

The transition function $t:\;S\times A\to S\cup s_{end}$ defines the next state based on the current state and action:


$$t\left(s_{t},a_{t}\right)=\;\left\{ \begin{array}{c} s_{end}\;\;\;\;\;\;\;\;\;\;\;\;\;\;\;\;\;\;\;\;\;\;\;\;\;\;\;\;\;\;\;\;\;\;\;\;\;\;\;\;\;\;\;\;\;\;\;\;\;\;\;\;\;if\;a_{t}\in A_{c}\\ s_{t+\text{1}}=\left(x_{t},y_{t},F_{t}\cup \{f_{i}\}\right)\;\;\;\;if\;a_{t}=f_{i}\;\in A_{f} \end{array}\right.\;$$ (2)

In this context, $s_{end}$ represents the terminal state. If the agent selects a feature, that feature $f_{i}$ is added to the current feature set $F_{t}$. The episode ends when the agent takes a classification action. It is important to note that the RL model operates in a discrete-time framework. The time index t in $s_{t}$ and $s_{t+\text{1}}$ reflects the sequential nature of feature acquisition decisions. At each time step, the agent chooses either to expand the feature set or to perform classification, with each action influencing future states and the resulting reward. This iterative process differentiates reinforcement learning from traditional supervised models where the feature set remains static.

The pseudocode in Algorithm 1 illustrates how the model uses feature extraction and prediction to implement the learning strategy. The method consists of randomizing the dataset, selecting actions aligned with the current strategy, executing these actions within the framework of the model, and modifying the strategy as feedback and state changes occur. The off-policy PPO algorithm is used to enhance policy training.

#### 3-1-1 Training.

To improve policy training, an off-policy PPO algorithm is integrated into the RL framework. Next, we detail the trust region policy optimization (TRPO) technique, designed to boost policy efficacy by refining a surrogate objective with on-policy data. Following this, we explore the PPO, which implements a clipped surrogate objective to limit the large policy updates typically seen in TRPO. Finally, we describe the off-policy PPO method, combining elements from TRPO and PPO.

***Trust region policy optimization*.** In traditional RL, the goal is to develop a policy, π, that maximizes the cumulative discounted reward starting from the initial state, as described below [18]:


$$\eta (\pi )=E_{s_{\text{0}},a_{\text{0}},\ldots }\left[R_{\text{0}}\right]=E_{s_{\text{0}},a_{\text{0}},\ldots }[\sum {\mathrm{\backslash nolimits}}_{t=\text{0}}^{\infty }\gamma^{t}r(s_{t},a_{t})$$ (3)

Algorithm 1: Pseudo-code for training the model.

Input: Training data Data = $\left\{\left(x_{\text{1}},\;y_{\text{1}}\right),\;\left(x_{\text{2}},\;y_{\text{2}}\right),\;\ldots ,\;\left(x_{T},\;y_{T}\right)\right\}$, learning rate α, maximum iteration E

Set initial values for policy parameters θ and $\theta_{old}$

Set initial values for value function parameters ϕ

Set initial advantage estimator A

Create initial replay memory B

Initialize action-value function Q with random weights

For e=1 to E do:

 ShuffleData

 For t=1 to T do:

  Choose the action $a_{t}$ with a probability of ε

  Set the state $s_{\text{1}}$

  If $a_{t}$ is a feature then:

   Execute the action $a_{t}\;$in the emulator

   Calculate the reward $r_{t}$ using Equation 1

   Identify the subsequent state${\;s}_{t+\text{1}}$ fromData 

   Record the transition ($s_{t}$,$\;a_{t}$,$\;r_{t}$,$\;s_{t+\text{1}},\mu (\cdot |s_{t})$) inB

   Compute the advantage $A(s_{t},\;a_{t})$ using the value function with parameters ϕ

   Update π to maximize the objective function in Equation 16:

    $\theta \;\leftarrow \;\theta_{old}\;+\;\alpha \nabla_{\theta }\;L_{Off-Policy\;PPO}^{CLIP}(\pi ).$

  If $a_{t}$ is a subject number then:

   Execute $a_{t}\;$in the emulator

   Calculate the reward $r_{t}$ using Equation 1

   Identify the subsequent state${\;s}_{t+\text{1}}$ fromData 

   Record the transition ($s_{t}$,$\;a_{t}$,$\;r_{t}$,$\;s_{t+\text{1}},\mu (\cdot |s_{t})$) inB

Initially, $s_{\text{0}}$ is drawn from a starting state distribution $\rho_{\text{0}}$. At any given time t, the variables $a_{t}$ and $s_{t}$ represent the action taken and the current state, respectively. These are determined by the policy $\pi (a_{t}|s_{t})$ and the probabilities of moving to the next state $P(s_{t+\text{1}}{|s}_{t},a_{t})$. γ is the discount factor used to prioritize immediate rewards over distant ones. The objective is to improve the efficiency of the policy (η(π)). To accomplish this, the TRPO method uses on-policy data. It seeks to improve policy effectiveness by optimizing a surrogate objective function within a defined limit for the Kullback-Leibler (KL) divergence [18]:


$$\underset{\pi }{\text{max}}E_{s\sim \rho_{\pi_{\theta_{old}}},\;\;a\;\in \;\pi_{\pi_{old}}}[\frac{\pi_{\theta }\left(a|s\right)}{\pi_{\theta_{old}}\left(a|s\right)}A_{\pi_{old}}(s,a)\backslash ]$$ (4)

Subject to


$$E_{s\sim \rho_{\pi_{old}}}\left[D_{KL}(\pi_{old}(.|s)||\;\pi (.|s))\right]\leq \delta$$ (5)

Here, $\pi_{old}$ represents the existing policy, and δ establishes the threshold for allowable divergence. The formula $D_{KL}(\pi_{old}(.|s)||\;\pi (.|s))$ measures the KL divergence, showing the degree to which the new policy π differs from the previous policy $\pi_{old}$ at any given state s. The term $\rho_{\pi_{old}}$ represents the probability distribution over states, starting from the initial state $s_{\text{0}}$ and following the previous policy $\pi_{old}$, calculated as $\rho_{\pi_{old}}(s)=\sum_{t=\text{0}}^{\infty }\gamma^{t}P(s_{t}=s|s_{\text{0}},\pi_{old})$. Without this divergence constraint, enhancing the surrogate objective function could lead to significant changes in the policy.

***Proximal policy optimization***. To prevent significant policy changes, PPO uses a modified surrogate objective that it aims to maximize. This modified objective, the clipped surrogate objective within PPO, is structured as follows [18]:


$$L_{PPO}^{CLIP}=E_{s\sim \rho_{\pi_{old},\;\;\;a\sim \pi_{old}}}[\text{min}(\frac{\pi \;\left(a|s\right)}{\pi_{old}\left(a|s\right)}A_{\pi_{old}}(s,a),clip(\frac{\pi \left(a|s\right)}{\pi_{old}\left(a|s\right)},\text{1}-\in ,\text{1}+\in )A_{\pi_{old}}(s,a))]$$ (6)

The variable ε represents a small positive value selected to balance maintaining policy stability and encouraging exploration. The advantage function $A_{\pi_{old}}(s,a)$ evaluates the extra gain from taking a specific action a in state s under the policy $\pi_{old}$, compared to the average result of all possible actions in the same state under that policy. This essentially determines whether an action performs better or worse than what is typically expected under that policy. In this context, clipping is a technique used to curb overly aggressive updates to the policy during training, thereby fostering more stable progress. This technique is further explained below [18]:


clip(x, a, b) = max(a, min(b, x)) (7)

Here, x is the parameter we adjust, with a and b defining the lower and upper limits of the allowable range for x. It is important to note that the clipped surrogate objective, detailed in Equation 6, is designed to prevent overly large updates to the policy by imposing penalties on changes that make the ratio $\frac{\pi \left(a|s\right)}{\pi_{old}\left(a|s\right)}\;$ stray far from 1. However, a significant challenge for PPO is its reliance on on-policy data, which increases sample complexity due to the limited use of off-policy data, necessitating frequent interactions between the agent and the environment [58].

***Off-policy PPO*.** This research presents the off-policy PPO, which improves data utilization by leveraging insights from previous interactions and diverse policy choices [59]. Contrary to the conventional on-policy PPO that relies on contemporary data for optimal performance, off-policy PPO gains advantages from a broader and more heterogeneous dataset. For example, in credit risk prediction for listed companies, off-policy PPO can analyze historical data from numerous corporate financial statements and market behaviors to forecast potential defaults. This approach enables the model to learn from vast arrays of past corporate financial outcomes and adapt to recognize patterns indicating fiscal stress or stability, which may not be evident in the current data alone. By utilizing this broader dataset, off-policy PPO can effectively predict credit risk by identifying subtle changes in financial indicators that precede the downturn of a company, thus offering a more robust and preemptive risk assessment strategy.

Off-policy PPO addresses the optimization challenge by enhancing a surrogate objective function utilizing off-policy data, similar to the method used in off-policy TRPO [18]:


$$\underset{\pi }{max}E_{s\sim \rho_{\mu },\;\;a\in \mu }\left[\frac{\pi \left(a|s\right)}{\mu \left(a|s\right)}A_{\pi_{old}}(s,a)\right]$$ (8)

Subject to:


$${\overset{―}{D}}_{KL}^{\rho_{\mu },sqrt}\left(\mu ,\pi_{old}\right){\overset{―}{D}}_{KL}^{\rho_{\mu },sqrt}\left(\pi_{old},\pi \right)+{\overset{―}{D}}_{KL}^{\rho_{\mu }}(\pi_{old},\pi )\leq \delta$$ (9)

Where


$$\rho_{\mu }(s)=\sum {\mathrm{\backslash nolimits}}_{t=\text{0}}^{\infty }\gamma^{t}P(s_{t}=s|s_{\text{0}},\mu )$$ (10)

$${\overset{―}{D}}_{KL}^{\rho_{\mu }}\left(\pi_{old},\pi \right)=E_{s\sim \rho_{\mu }}[D_{KL}\left(\pi_{old}\left(.|s\right)\;||\;\pi \left(.|s\right)\right)]$$ (11)

$${\overset{―}{D}}_{KL}^{\rho_{\mu },sqrt}\left(\mu ,\pi_{old}\right)=E_{s\sim \rho_{\mu }}[\sqrt{D_{KL}\left(\mu \left(.|s\right)\;||\;\pi_{old}\left(.|s\right)\right)}]$$ (12)

$${\overset{―}{D}}_{KL}^{\rho_{\mu },sqrt}\left(\pi_{old},\pi \right)=E_{s\sim \rho_{\mu }}[\sqrt{D_{KL}\left(\;\pi_{old}\left(.|s\right)\;||\;\pi \left(.|s\right)\right)}]$$ (13)

Here, μ represents the behavior policy. Without the restrictions noted in Equation 9, aiming to enhance the surrogate function with off-policy data (as described in Equation 8) can result in substantial modifications in the policy. To address this potential issue, we use the PPO clipping technique, adjusting the surrogate objective accordingly [18]:


$$L_{\mu }(\pi )=E_{s\sim \rho_{\mu },\;\;a\in \mu }[\frac{\pi \left(a|s\right)}{\mu \left(a|s\right)}A_{\pi_{old}}(s,a)]$$ (14)

Using $L_{\mu }(\pi )$ from Equation 14, we define the clipped surrogate objective for off-policy data as follows [18]:


$${\overset{―}{L}}_{\mu }(\pi )=E_{s\sim \rho_{\mu },\;\;a\in \mu }[\text{min}(\frac{\pi \left(a|s\right)}{\mu \left(a|s\right)}A_{\pi_{old}}(s,a),clip(\frac{\pi \left(a|s\right)}{\mu \left(a|s\right)},\text{1}-\in ,\text{1}+\in )A_{\pi_{old}}(s,a))]$$ (15)

The ratio $\frac{\pi \left(a|s\right)}{\mu \left(a|s\right)}$ frequently falls beyond the range of 1 − ε to 1 + ε. Consequently, the policy π(a|s) generally remains unaltered when optimizing the clipped surrogate objective. To mitigate this inertia, the limits of the clipped objective, from (1 − ε) to (1 + ε), are recalibrated in Equation 15 by integrating a correction term $\frac{\pi_{\theta_{i}}\left(a|s\right)}{\mu \left(a|s\right)}$ [18]:


$$L_{Off-Policy\;PPO}^{CLIP}(\pi )=E_{s\sim \rho_{\mu },\;\;a\in \mu }[\text{min}[\frac{\pi \left(a|s\right)}{\mu \left(a|s\right)}A_{\pi_{old}}(s,a),clip\left(\frac{\pi \left(a|s\right)}{\mu \left(a|s\right)},\frac{\pi_{old}\left(a|s\right)}{\mu \left(a|s\right)}(\text{1}-\in ),\frac{\pi_{old}\left(a|s\right)}{\mu \left(a|s\right)}(\text{1}+\in )\right)A_{\pi_{old}}(s,a)]]$$ (16)

**Fig 2:**
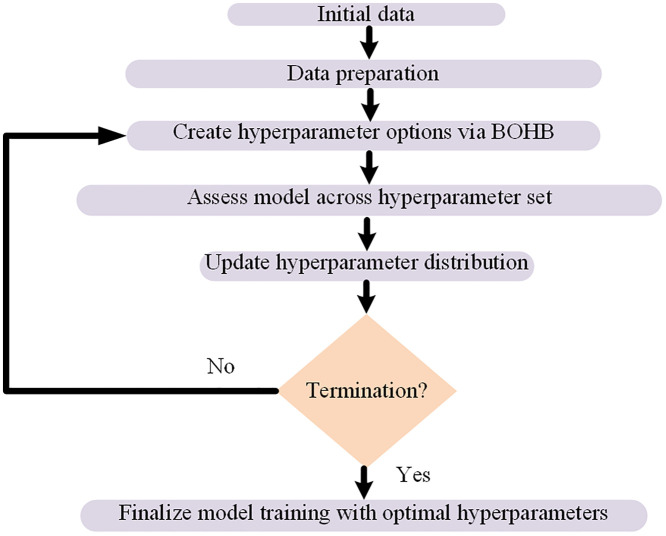
Optimization procedure of the predictive credit risk model utilizing off-policy PPO.

Fig 2 outlines the method to optimize the proposed credit risk prediction model using off-policy PPO. The approach is segmented into seven distinct steps, described below:

- The first step establishes an optimization framework that utilizes the MLP network to process past data and prior policies, enhancing its predictive performance. This arrangement seeks to heighten the effectiveness of the model by integrating insights from previous data, as explained in Equation 8.
- The second step introduces a constrained surrogate objective to regulate policy updates, limiting permissible changes. This provision guarantees that the adjustments of the method are tempered and its strength is maintained, as indicated in Equation 14.
- The third step involves enforcing a clipping strategy to maintain updates within a predetermined safe boundary, confirming that policy modifications remain between 1−∈ and 1+∈, as depicted in Equation 15. This safeguard is critical to avert drastic fluctuations that might impair the stability of the model.
- The fourth step tailors the clipped surrogate objective to accommodate off-policy data, allowing the MLP to utilize historical data in the learning regimen without the risk of overfitting, applying Equation 15 to past exchanges.
- The fifth step verifies that the policy proportion between fresh and existing policies remains within the established bounds of 1−∈ to 1+∈. Deviations outside this range may indicate overly aggressive updates.
- The sixth step employs corrective measures to realign policy updates to their desired levels if they stray from the specified range, as detailed in Equation 16. This adjustment is critical for the MLP network to prevent drastic alterations based on potentially obsolete data.
- The final step involves updating the policy, represented by the MLP network, based on the refined clipped objective. This cycle is reiterated until the model reaches a steady, optimized state. Through this iterative process, the MLP network continually enhances its accuracy and feature discernment in credit risk prediction.

### 3.2 Hyperparameter optimization

Hyperparameter optimization is crucial for improving the performance of machine learning algorithms [56,60]. Consider a machine learning algorithm as a function g: χ→R, where χ includes all possible hyperparameters, each combination represented by x within χ. Hyperparameter optimization aims to find the best set of hyperparameters, $x^{*}$, that minimizes the function g(x). However, directly observing g(x) is often impractical for many machine learning algorithms because of their inherent variability and uncertainties. It is generally assumed that the function can only be observed through noisy evaluations:


y(x) = g(x) + ∈ (17)

where ε represents a noise component conforming to a normal distribution characterized by a mean of zero and variance $\sigma_{noise}^{\text{2}}$.

#### 3-2-1 Bayesian optimization.

Bayesian optimization (BO) is an advanced technique for fine-tuning complex black-box functions, which are typically expensive and challenging to assess. This method is particularly valuable when these functions lack straightforward analytic forms and require significant computational resources to evaluate. A primary application of BO is in the optimization of hyperparameters for complex machine-learning models. BO focuses on creating a probabilistic model that predicts the efficacy of various inputs, thus aiding in pinpointing the most promising points for evaluation.

Bayesian optimization (BO) progresses iteratively, developing a probabilistic model named p(g|D) to estimate the target function g using Gaussian Processes. It uses accumulated data from a dataset noted as $D\;=\{\left(x_{\text{0}},y_{\text{0}}\right),\left(x_{\text{1}},y_{\text{1}}\right),\ldots ,(x_{i-\text{1}},y_{i-\text{1}}\}$. An essential element of this model is the acquisition function a: X→R, designed to balance exploring new areas and exploiting known advantageous locations, reflecting the most recent data insights from p(g|D).

The iterative process includes the following steps

- Choosing the point $x_{select}$ where the acquisition function reaches its peak, characterized as:

$$x_{select}=\text{arg}{\text{max}}_{x\;\in \;\chi }a(x)\;\;$$ (18)

- Establishing the precise measurement of the objective function at the designated point is illustrated below:

$$y_{select}=g\left(x_{select}\right)+\in \;\;\;\;$$ (19)

- Adding the latest observation to the existing dataset D, thus updating the model with new data:

$$D\;=\;D\;\cup (x_{select},y_{select})$$ (20)

- Each cycle incrementally builds upon the previous one, continuously improving the accuracy and ability of the model to identify the optimal observation, referred to as $x_{best}$. The fundamental goal is to advance toward:

$$x_{best}=\text{arg}{\text{min}}_{x\;\in \;D}g(x)\;\;\;\;$$ (21)

where the system precisely pinpoints the optimal point to minimize the objective function, achieving the optimization goals. This continuous modeling, evaluation, and enhancement cycle is crucial to BO, making it a successful method for addressing complex optimization challenges through deliberate decision-making and computational effectiveness.

#### 3.2.2 Hyperband.

Hyperband (HB) is a strategy that optimizes resource use in hyperparameter tuning by focusing on exploration. It introduces a new approach to evaluating setups by applying hyperparameter optimization principles centered on an early-stopping mechanism. This approach quickly identifies the best hyperparameters across various settings, enhancing the efficiency and cost-effectiveness of hyperparameter space exploration and thereby speeding up the identification and prioritization of the best configurations, ultimately improving the overall optimization process.

#### 3.2.3 BOHB hyperparameter optimization.

BOHB is an advanced hyperparameter optimization algorithm that synergistically combines the strengths of BO and HB. Traditional BO methods, like the tree-structured Parzen estimator (TPE), model the performance of hyperparameter configurations to guide the search for optimal settings. BOHB enhances this method by employing a single kernel density estimator that considers all hyperparameters simultaneously. This approach facilitates a deeper understanding of how different hyperparameters interact with each other.

In the BOHB framework, the random sampling strategy of Hyperband is replaced with model-based sampling. This means that, instead of selecting configurations at random, BOHB uses the kernel density estimator (KDE) model to propose promising configurations based on past evaluations. These configurations are then evaluated by the successive halving strategy used in Hyperband. This strategy allocates more resources to configurations that perform better during early evaluations.

By integrating model-based sampling with adaptive resource allocation, BOHB efficiently navigates the hyperparameter space. It balances trying new configurations with focusing on those that have already shown good performance. This balance makes the algorithm well-suited for situations with limited computing power and complex sets of hyperparameters. This approach allows for the discovery of high-performing configurations with fewer evaluations compared to traditional methods. The pseudo-code of BOHB is given in Algorithm 2 in Appendix A in S1 Appendix.

### 3.3 Overall algorithm

**Fig 3:**
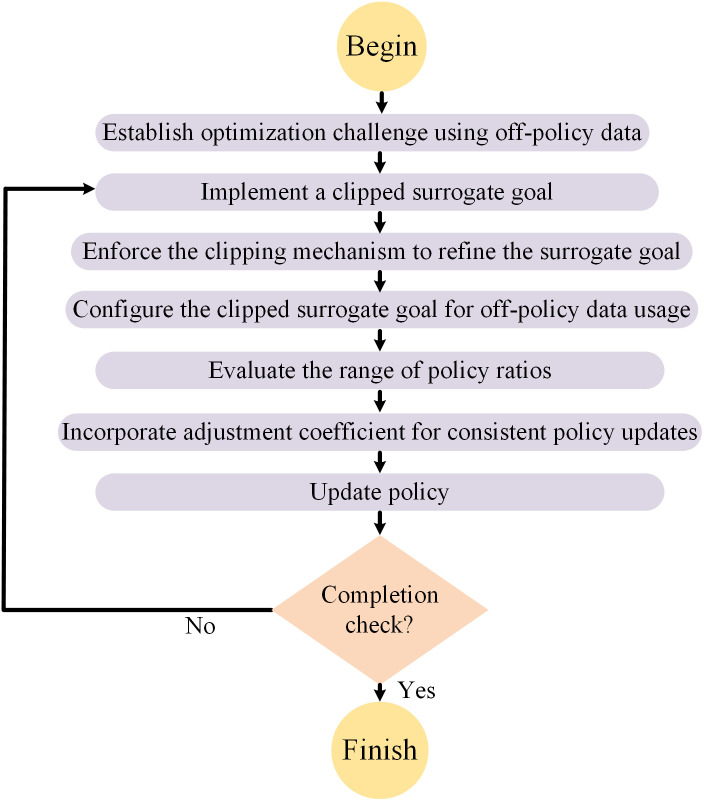
Overall flowchart of the proposed model.

Fig 3 visually outlines the workflow of the proposed model, detailing each step from initial data input to final classification. The process begins with the input dataset, which serves as the foundation for all subsequent steps. Initially, the data undergoes preprocessing to ensure the modeling relies on clean and appropriately scaled data. The first preprocessing step is the removal of missing values, which is essential for preventing biased or inaccurate results in predictive modeling. Removing missing data ensures that all data points are complete, thereby eliminating potential errors from incomplete data and enhancing the reliability and consistency of the model. After removing missing values, the data is normalized using min-max scaling, adjusting feature scales to a 0–1 range. Such scaling benefits handling features with wide-ranging magnitudes and units, ensuring that no single feature dominates due to its scale. This normalization makes our model robust, helping it perform uniformly across all data features. These preprocessing steps prove effective as they prepare the data for efficient analysis. Eliminating missing values guarantees that our model trains on complete cases, improving prediction accuracy. Normalization prevents the model from being biased toward larger-scale features, thereby boosting predictive performance. These steps lay a solid foundation for the later stages of feature selection and model training, ultimately leading to more reliable and interpretable results in credit risk prediction.

After data preparation, we define the boundaries for hyperparameter search, specifying possible values for each hyperparameter as shown in Table 2. The BOHB method then generates various hyperparameter candidates within these boundaries. Employing Bayesian optimization, BOHB focuses on promising areas and uses Hyperband to manage resources efficiently, which is crucial for evaluating each setup under limited resources. Each hyperparameter set configures the model, which is then evaluated using accuracy metrics to determine its effectiveness in addressing the optimization challenges of the model. Following each evaluation, BOHB updates its model of the hyperparameter space to improve predictions of which settings are likely to lead to better outcomes. This iterative process of generating, assessing, and updating hyperparameters continues for 20 iterations, culminating in the selection of the optimal hyperparameter configuration that best trains the model.

**Table 2:** Hyperparameters and their ranges for optimization in the credit risk prediction method.

Name	Intervals	Optimal value
Batch size	[8, 512]	CSMAR (65 and MorningStar (72
Epoch	[32, 1024]	CSMAR (269) and MorningStar (282)
Learning rate (α)	[0, 1]	CSMAR (0.08) and MorningStar (0.02)
Activation	{Tanh, Leaky ReLU, Linear, ReLU, Sigmoid}	CSMAR (ReLU) and MorningStar (ReLU)
Dropout rate	[0, 1]	CSMAR (0.4) and MorningStar (0.6)
Layers in MLP	[1,8]	CSMAR (3) and MorningStar (5)
Hidden size	[8, 1024]	CSMAR (35) and MorningStar (48)
Balancing parameter (λ)	[0, 1]	CSMAR (0.2) and MorningStar (0.4)

## 4. Empirical evaluation

The following section presents a comprehensive analysis of the dataset, including its properties and its integration into our research. Following this, a detailed review of the metrics used to evaluate the effectiveness of our models will be conducted. We will specify the benchmarks and standards used to gauge the performance of our models. This segment concludes with the results of our investigation, emphasizing the key insights derived from analyzing our data and evaluating our models. Additionally, we will discuss the relevance of these results, particularly in relation to the objectives of our study.

### 4.1 Datasets

To evaluate the proposed credit risk prediction model, we use the CSMAR and MorningStar databases [1], which were chosen for several compelling reasons. One of the most important reasons is their reflection on real-world economic and financial conditions. Both databases offer extensive, up-to-date, and comprehensive data sets representing actual market behaviors and trends, providing a solid empirical foundation for testing. CSMAR includes detailed information specific to the Chinese market, making it invaluable for assessing models within this context. MorningStar provides a broad spectrum of global financial data, ensuring that our model can be assessed against diverse investment scenarios and market conditions. These databases not only enhance the validity of our model by exposing it to realistic conditions but also ensure that the results are robust, reliable, and applicable to academic research and practical financial analysis [41].

CSMAR is a detailed and research-oriented economics and finance database designed to align with the high standards of globally recognized databases such as CRSP, Compustat, TAQ, and Thomson from the University of Chicago, explicitly tailored to include the unique socio-economic conditions of China. Over two decades of consistent enhancement and development have broadened the scope of the CSMAR database to encompass areas such as the green economy, stocks, corporate data, the securities and futures markets of China, foreign exchange, macroeconomics, and various sectors pertinent to economic and financial research. It now boasts over 200 databases, 4,000 + tables, and 60,000 + fields, featuring data sorted by time, codes, and other indices for graphical representation within the database. Data can also be exported in formats such as CSV for versatile use. The stock exchange information within CSMAR has become an indispensable resource for investment and empirical research.

The Morningstar database provides investors with expert financial insights through user-friendly analyses, ratings, and tools, including features such as Ratings, Investment Style Box, and Category Ratings. These resources are designed to be straightforward yet professional, helping investors make well-informed decisions. The data extracted from the Morningstar database is readily accessible and reproducible, facilitating the swift retrieval of financial information. With data on roughly 500,000 investment products, the Morningstar database is extensively utilized across various scholarly fields. This study leverages the comprehensive data available from the database on public companies.

To evaluate the generalizability of the proposed model, we utilized the KMV default dataset [1], along with two publicly available datasets: GMSC [61] and UCICCD [62]. The KMV default dataset is developed and maintained by Moody Analytics. It provides a comprehensive collection of historical credit data on firms from various industries and regions. It captures rich, time-series data on corporate defaults, recovery rates, and detailed financial indicators, including balance sheet ratios, equity volatility, and capital structure variables. The dataset spans over 40 years, covering firm-level observations from the early 1980s to the present, and includes more than 400,000 firm-year records across over 20,000 companies in both developed and emerging markets. Each record typically includes monthly or quarterly financial data, market capitalization, leverage ratios, interest coverage, and industry-specific risk metrics. The Moody KMV model calculates the expected default frequency (EDF) by combining market-based indicators such as equity value and volatility with accounting-based metrics. This process yields default probabilities over time horizons such as 1 year and 3 years. The EDF scores are widely used by global banks, insurance firms, and regulators as a standard input for internal rating systems, portfolio risk management, capital adequacy modeling, and compliance with frameworks such as Basel III. The breadth, depth, and temporal resolution of the KMV dataset make it particularly valuable for validating advanced credit risk models on firm-level default prediction tasks.

The GMSC dataset, publicly released on Kaggle, comprises financial profiles of 150,000 individual loan applicants in the United States. It includes 11 numerical features that describe credit behavior. These features cover monthly income, debt-to-income ratio, delinquency counts over 30, 60, and 90 days, and use of revolving credit. The target variable is binary, indicating whether an individual experienced a serious delinquency, typically defined as being more than 90 days late within a two-year period. Approximately 6.68% of the individuals in the dataset fall into the default class, resulting in a realistically imbalanced distribution that is representative of real-world credit data. This dataset is widely used in academic and industrial research due to its size, realism, and imbalanced class distribution. It is especially valuable for evaluating credit risk models that address class imbalance and select important financial features.

The UCICCD dataset, hosted by the UCI Machine Learning Repository and originally compiled by the Taiwan Economic Journal, comprises 30,000 records of credit card customers in Taiwan. Each record contains 23 features, including demographic details, credit limits, and financial behavior, such as repayment and billing patterns history, over six months. The target variable is a binary indicator showing whether a customer defaulted on their payment in the subsequent month. Notably, approximately 22.1% of the customers in the dataset are labeled as defaulters, resulting in a moderately imbalanced distribution that reflects real-world credit risk. This dataset is widely used because it is complete, has no missing values, and includes detailed sequential financial data. These qualities make it ideal for testing models that predict individual default risk.

### 4.2 Metrics

This article evaluates the proposed credit risk prediction model using accuracy, F-measure, geometric mean (G-means), and area under the curve (AUC) metrics. Each metric is selected for its comprehensive assessment capabilities, which are essential in credit risk prediction. Accuracy directly calculates the effectiveness of the method across all predictions. The F-measure combines precision and recall and is crucial for assessing the balance between accurately identifying positive cases and minimizing false positives. G-means is chosen for its utility in evaluating models within imbalanced datasets, which are common in credit risk environments where defaults are rarer than non-defaults. The AUC offers an extensive evaluation of the capacity of the model to distinguish between classes at varying thresholds, which is crucial for appraising the sophisticated decision-making needed in financial risk evaluations. Together, these metrics provide a comprehensive assessment, highlighting both the efficiency and equity of the predictive model.

Formally, the metrics of accuracy, F-measure, and G-means are described as follows:


$$Accuracy=\frac{True\;Positives\;(TP)+True\;Negatives\;(TN)}{Total\;observations}\;\;\;\;$$ (22)

$$F-Measure\;(F\text{1}\;Score)=\text{2}\times \frac{Precision+Recall}{Precision+Recall}\;\;\;\;$$ (23)

$$G-means=\;\sqrt{Recall\times Specificity}\;\;$$ (24)

where


$$Precision=\frac{TP}{TP+False\;Positives\;(FP)}\;\;\;\;$$ (25)

$$Recall=\frac{TP}{TP+False\;Negatives\;(FN)}\;\;\;\;$$ (26)

$$Specificity=\frac{TN}{TN+FP}\;\;\;\;\;\;\;\;\;$$ (27)

### 4.3 Main results

All experiments were executed using an NVIDIA graphics processing unit (GPU) with compute unified device architecture (CUDA) support, which enabled efficient acceleration of machine and deep learning tasks. The study was conducted on a 64-bit Windows operating system, utilizing an Intel Core i7 processor with 64 gigabytes (GB) of random-access memory (RAM) to ensure adequate computational power for intensive data processing and model training. The models were implemented using Python programming, primarily employing libraries such as PyTorch for building and training the neural network models and Scikit-learn for traditional machine learning algorithms. All experiments were conducted using Python 3.9 and PyTorch 1.13.1 with the random seed set to 42. We utilized the stable baselines library of Python for the off-policy PPO algorithm and the HpBandSter library for the BOHB algorithm. This setup allowed for efficient execution of reinforcement learning procedures and hyperparameter optimization tasks. Data manipulation and preparation were also handled using Pandas and NumPy libraries, which provided robust tools for managing the datasets sourced from CSMAR and MorningStar. The software environment was managed via Anaconda, which simplified package management and deployment, ensuring that all dependencies were consistently maintained across the computational setup. This combination of advanced hardware and sophisticated software frameworks enabled the precise and effective evaluation of the proposed credit risk prediction models.

To assess the reliability and precision of our models, we implement a 5-fold stratified cross-validation method. This technique is particularly advantageous in credit risk prediction because it maintains the proportion of different classes within each fold of the dataset. In the context of credit risk, where data may be imbalanced between classes, stratified cross-validation ensures that each fold accurately represents the overall population. This helps avoid biases in model training and validation phases, which can occur if one class is overrepresented or underrepresented in any folds. Additionally, this method provides a robust measure of model performance by testing the model across multiple, varied subsets of data, leading to more generalizable and reliable predictive performance across different scenarios. This approach enhances confidence in the ability of the model to accurately and consistently predict outcomes in practical, real-world applications where the distribution of outcomes is crucial.

In the evaluation phase, our model was extensively compared with eight machine learning models, including XGBoost [11], PSO-XGBoost [6], SW-SVDD [27], LightGBM-PCA-SMOTEENN [28], LightGBM [31], TSC-SVM [32], ES-LqSVM [34], SMPSO-NSGA-FS [36], and twelve deep learning models, HDNN [37], CNN-LSTM [1], CNN-XGBoost [38], CNN-BiLSTM [39], kNN–GNN [40], URP-CNN [41], GAN-SynthRisk [48], TabTransformer-WLoss [49], TabTransformer-LIME [50], GAN-Oversample [51], TCN-DilateFormer [54], DAMEL [55]. Additionally, we evaluated the impact of removing critical elements such as feature selection (FS), off-policy PPO, and hyperparameter optimization (HO) from our model to understand their contributions to its overall performance. The outcomes of these comparisons, conducted on the CSMAR and MorningStar datasets, are detailed in Tables 3 and 4.

**Table 3:** Performance comparison of the proposed model against advanced and ablated studies using the CSMAR dataset.

Model	Accuracy	F-measure	G-means	AUC
XGBoost [11]	73.471 ± 0.041	72.970 ± 0.088	73.703 ± 0.049	0.671 ± 0.015
PSO-XGBoost [6]	73.765 ± 0.085	73.619 ± 0.054	74.334 ± 0.074	0.687 ± 0.060
SW-SVDD [27]	74.495 ± 0.023	74.807 ± 0.063	75.528 ± 0.051	0.713 ± 0.083
LightGBM-PCA-SMOTEENN [28]	82.795 ± 0.043	82.721 ± 0.063	83.331 ± 0.095	0.774 ± 0.020
LightGBM [31]	74.063 ± 0.006	74.143 ± 0.042	74.866 ± 0.065	0.704 ± 0.028
TSC-SVM [32]	74.910 ± 0.043	74.848 ± 0.078	75.673 ± 0.049	0.710 ± 0.054
ES-LqSVM [34]	75.610 ± 0.026	74.318 ± 0.076	76.146 ± 0.081	0.727 ± 0.081
SMPSO-NSGA-FS [36]	76.390 ± 0.080	75.822 ± 0.016	76.673 ± 0.039	0.739 ± 0.098
HDNN [37]	83.331 ± 0.069	83.045 ± 0.003	83.655 ± 0.034	0.790 ± 0.042
CNN-LSTM [1]	84.187 ± 0.027	83.813 ± 0.070	84.425 ± 0.032	0.801 ± 0.079
CNN-XGBoost [38]	84.510 ± 0.079	84.360 ± 0.013	84.961 ± 0.058	0.811 ± 0.055
CNN-BiLSTM [39]	85.434 ± 0.011	85.147 ± 0.071	85.735 ± 0.025	0.819 ± 0.080
kNN–GNN [40]	86.048 ± 0.079	85.961 ± 0.049	86.544 ± 0.038	0.832 ± 0.008
URP-CNN [41]	82.478 ± 0.075	81.763 ± 0.076	81.378 ± 0.016	0.751 ± 0.009
GAN-SynthRisk [48]	80.035 ± 0.080	80.836 ± 0.031	81.598 ± 0.070	0.727 ± 0.085
TabTransformer-WLoss [49]	83.742 ± 0.009	82.483 ± 0.002	83.248 ± 0.059	0.762 ± 0.042
TabTransformer-LIME [50]	84.045 ± 0.008	84.233 ± 0.066	84.957 ± 0.036	0.768 ± 0.097
GAN-Oversample [51]	84.768 ± 0.024	84.884 ± 0.080	85.970 ± 0.001	0.772 ± 0.045
TCN-DilateFormer [54]	85.093 ± 0.044	85.522 ± 0.039	86.201 ± 0.081	0.778 ± 0.058
DAMEL [55]	87.572 ± 0.076	87.826 ± 0.094	88.831 ± 0.008	0.795 ± 0.088
Proposed w/o FS	83.276 ± 0.093	82.565 ± 0.073	82.155 ± 0.009	0.762 ± 0.087
Proposed w/o off-policy PPO	84.134 ± 0.098	83.254 ± 0.063	82.854 ± 0.060	0.769 ± 0.020
Proposed w/o HO	85.179 ± 0.036	84.429 ± 0.008	83.157 ± 0.060	0.779 ± 0.091
Proposed	92.478 ± 0.075	90.763 ± 0.076	91.378 ± 0.016	0.889 ± 0.005

**Table 4:** Performance comparison of the proposed model against advanced and ablated studies using the MorningStar dataset.

Model	Accuracy	F-measure	G-means	AUC
XGBoost [11]	70.812 ± 0.051	70.803 ± 0.039	71.650 ± 0.041	0.628 ± 0.034
PSO-XGBoost [6]	71.215 ± 0.004	71.516 ± 0.071	72.367 ± 0.047	0.634 ± 0.009
SW-SVDD [27]	72.905 ± 0.008	72.628 ± 0.021	73.455 ± 0.022	0.653 ± 0.085
LightGBM-PCA-SMOTEENN [28]	73.842 ± 0.011	73.469 ± 0.047	74.294 ± 0.061	0.663 ± 0.055
LightGBM [31]	72.053 ± 0.075	72.203 ± 0.021	73.019 ± 0.052	0.640 ± 0.052
TSC-SVM [32]	72.812 ± 0.038	72.803 ± 0.058	73.650 ± 0.035	0.645 ± 0.073
ES-LqSVM [34]	73.660 ± 0.035	73.604 ± 0.022	74.430 ± 0.007	0.648 ± 0.068
SMPSO-NSGA-FS [36]	74.053 ± 0.047	74.089 ± 0.035	75.905 ± 0.010	0.658 ± 0.081
HDNN [37]	79.567 ± 0.050	78.611 ± 0.036	79.435 ± 0.030	0.762 ± 0.057
CNN-LSTM [1]	82.376 ± 0.095	80.204 ± 0.064	81.009 ± 0.093	0.770 ± 0.013
CNN-XGBoost [38]	83.042 ± 0.085	80.585 ± 0.017	81.386 ± 0.082	0.786 ± 0.036
CNN-BiLSTM [39]	83.486 ± 0.037	81.393 ± 0.055	82.161 ± 0.001	0.803 ± 0.061
kNN–GNN [40]	84.112 ± 0.011	82.154 ± 0.094	82.925 ± 0.093	0.813 ± 0.069
URP-CNN [41]	84.444 ± 0.070	82.746 ± 0.099	83.535 ± 0.026	0.825 ± 0.004
GAN-SynthRisk [48]	82.744 ± 0.008	82.344 ± 0.003	83.168 ± 0.050	0.809 ± 0.067
TabTransformer-WLoss [49]	83.295 ± 0.098	82.260 ± 0.022	83.065 ± 0.045	0.827 ± 0.054
TabTransformer-LIME [50]	84.058 ± 0.095	84.092 ± 0.009	85.877 ± 0.020	0.831 ± 0.081
GAN-Oversample [51]	84.440 ± 0.059	84.618 ± 0.052	85.919 ± 0.059	0.833 ± 0.098
TCN-DilateFormer [54]	85.135 ± 0.011	84.167 ± 0.070	85.961 ± 0.100	0.838 ± 0.025
DAMEL [55]	85.440 ± 0.082	84.272 ± 0.006	85.137 ± 0.085	0.840 ± 0.021
Proposed w/o FS	80.199 ± 0.078	79.928 ± 0.039	79.761 ± 0.061	0.742 ± 0.023
Proposed w/o off-policy PPO	81.844 ± 0.050	80.505 ± 0.063	79.303 ± 0.009	0.758 ± 0.032
Proposed w/o HO	82.734 ± 0.040	81.954 ± 0.092	80.773 ± 0.075	0.762 ± 0.083
Proposed	89.692 ± 0.064	86.358 ± 0.074	87.123 ± 0.076	0.863 ± 0.085

For the CSMAR dataset, performance improves as we progress from basic machine learning techniques, such as XGBoost, to more advanced deep learning approaches. The integration of PCA and SMOTEENN with LightGBM, known as LightGBM-PCA-SMOTEENN, significantly enhances the handling of credit risk data, achieving an accuracy of 82.795%. This marks a 9.32% improvement over the basic XGBoost model, demonstrating the effectiveness of these techniques in managing the imbalanced datasets often found in credit risk scenarios. Among traditional models, CNN-BiLSTM performs the best, achieving an accuracy of 85.434%. It captures both temporal and spatial dependencies by combining convolutional and bidirectional long short-term memory layers. Recent machine learning models, such as SMPSO-NSGA-FS (76.390%) and ES-LqSVM (75.610%), demonstrate modest improvements over classic SVM variants, highlighting the impact of advanced feature selection and optimization techniques. TSC-SVM also outperforms XGBoost, indicating the benefit of semi-supervised and clustering integration.

Deep learning models, such as kNN-GNN, which are based on graph neural networks, generally outperform traditional models. This model achieves the highest accuracy of 86.048% and effectively handles the complex structures found in financial datasets. Hybrid deep models like TabTransformer-LIME (84.045%) and TabTransformer-WLoss (83.742%) demonstrate the growing importance of interpretable architectures for tabular credit data. GAN-based approaches also show promise, with GAN-Oversample reaching 84.768% and GAN-SynthRisk achieving 80.035%, validating the capacity of GANs for tackling class imbalance. Among these, DAMEL achieves 87.572%, ranking as the best-performing deep learning model before the proposed one. Its strength lies in its multistage ensemble design and dynamic domain adaptation.

The proposed model still outperforms all others, achieving 92.478% accuracy, which is 4.91% higher than DAMEL. The proposed model also shows substantial improvements in F-measure, G-means, and AUC, highlighting its advanced predictive capabilities and effective management of imbalanced data. These results highlight the benefit of using off-policy PPO and hyperparameter optimization to capture complex financial patterns. The importance of each component becomes evident when they are removed. Without feature selection, accuracy drops to 83.276%. Without off-policy PPO, it falls to 84.134%. Without HO, it decreases to 85.179%. These figures highlight the significant impact of each component on the overall effectiveness of the model, with FS being the most influential, followed by off-policy PPO and HO.

For the MorningStar dataset, LightGBM-PCA-SMOTEENN emerged as the top performer among machine learning models, registering a 4.3% higher accuracy than the basic XGBoost model. This demonstrates the advantage of integrating PCA and SMOTEENN in managing the imbalanced datasets often encountered in credit risk scenarios. Among machine learning enhancements, SMPSO-NSGA-FS surpassed earlier models by reaching 74.053% accuracy, demonstrating the strength of optimization-based feature selection. Additionally, TSC-SVM and ES-LqSVM showed consistent gains over classic SVM structures, reflecting improvements from semi-supervised and adaptive norm strategies.

In deep learning models, URP-CNN exhibited the best performance, showing a 16.6% increase in accuracy over the basic HDNN model. This indicates that URP-CNN can manage spatial and feature relationships found in non-linear credit risk patterns. However, newer models have now outperformed URP-CNN. GAN-SynthRisk and TabTransformer-WLoss both exceeded 83%, showing how GAN-based oversampling and tabular transformers contribute to balanced learning. TabTransformer-LIME, GAN-Oversample, and TCN-DilateFormer further pushed accuracy above 84%. DAMEL currently holds the top deep learning performance with 85.440% accuracy, thanks to its multistage ensemble and domain adaptation techniques.

The proposed model achieved significant enhancements over the highest-performing state-of-the-art model, DAMEL, with improvements of 4.25% in accuracy, 2.09% in F-measure, 1.99% in G-means, and 2.3% in AUC. These metrics confirm that reinforcement learning and Bayesian optimization help the model learn from complex and imbalanced data. When key components, such as FS, off-policy PPO, and HO, were removed, the performance of the model declined, underscoring their substantial contributions. Specifically, removing FS resulted in a 10.63% drop in accuracy, eliminating off-policy PPO led to an 8.76% decrease, and the absence of HO caused a 7.78% reduction in accuracy. These impacts show that each component is essential for achieving optimal performance.

To determine the statistical significance of the superiority of our model over other models, we performed paired t-tests using data from the CSMAR and MorningStar datasets. The 95% confidence intervals were calculated using the standard error of the differences in paired sample means, assuming approximate normality of the performance differences. For the CSMAR dataset, our model significantly outperformed all others, with all metrics showing statistically significant p-values. For example, compared to DAMEL, the best-performing existing model from the CSMAR dataset, our model showed significant improvements with p-values of 0.0032 for accuracy, 0.0039 for F-measure, 0.0045 for G-means, and 0.0036 for AUC. These results confirm the effectiveness of enhancements, such as off-policy PPO and advanced hyperparameter optimization, tailored to the specific complexities of financial data in the CSMAR dataset. Similarly, for the MorningStar dataset, the proposed model outperformed the best existing model, DAMEL, with p-values of 0.0009 for accuracy, 0.0011 for F-measure, 0.0011 for G-means, and 0.0013 for AUC, indicating significant enhancements. All reported confidence intervals, such as [5.6%, 6.2%] for accuracy, were constructed at the 95% confidence level using these t-distribution-based calculations. The analyses for both datasets demonstrate the statistical significance, robustness, and adaptability of the improvements provided by the proposed model across various financial contexts. The consistent superiority across both datasets illustrates that the machine learning innovations within the model are effectively customized to address the specific challenges and complexities of each dataset, thereby enhancing the accuracy and generalizability of credit risk predictions. This highlights the potential of the proposed model to significantly enhance credit risk assessment technologies, thereby ensuring more reliable and informed decision-making in financial applications.

Table 5 presents a comparison of the computational efficiency of the proposed model with that of ten other leading models. The comparison includes runtime, GPU memory usage, floating point operation (FLOP), and inference time per sample (ITPS) on both the CSMAR and MorningStar datasets. The proposed model shows substantial improvements across all four metrics. On the CSMAR dataset, it reduces runtime by 27.8% compared to DAMEL (2,625 seconds vs. 3,637 seconds) and by 25.7% on MorningStar (2,756 seconds vs. 3,708 seconds). It also lowers GPU usage by 26.3% (from 21.6 GB to 29.3 GB) on CSMAR and 25.2% (from 22.9 GB to 30.6 GB) on MorningStar. FLOPs decrease by approximately 42.2% and 35.5%, respectively. The inference time per sample is 18.2 ms for CSMAR and 17.1 ms for MorningStar. This represents a more than 38% improvement in responsiveness compared to DAMEL. These reductions in computational demands confirm that the proposed model is highly efficient and suitable for real-time credit risk prediction in practical financial systems.

**Table 5:** Computational efficiency comparison of the models on the CSMAR and MorningStar dataset.

Model	Dataset							
	CSMAR				MorningStar			
	Runtime (s)	GPU (GB)	FLOP ($\times 10^{10}$)	ITPS (ms)	Runtime (s)	GPU (GB)	FLOP ($\times 10^{10}$)	ITPS (ms)
XGBoost [11]	2256	19.5	1.24	12.4	2385	21.5	1.32	13.2
PSO-XGBoost [6]	2585	18.4	1.02	10.2	2769	19.4	1.14	11.4
SW-SVDD [27]	1940	19.6	1.14	11.4	2158	21.4	1.42	14.2
LightGBM-PCA-SMOTEENN [28]	2435	17.0	0.86	8.6	2569	18.6	1.01	10.1
LightGBM [31]	2156	17.3	0.82	9.6	2244	20.6	0.96	8.2
TSC-SVM [32]	2315	17.7	1.02	10.2	2442	19.1	1.16	11.6
ES-LqSVM [34]	2207	20.1	1.42	14.2	2348	21.9	1.57	15.7
SMPSO-NSGA-FS [36]	2366	20.5	1.18	12.9	2466	21.8	1.29	11.8
HDNN [37]	2786	20.7	1.42	15.4	2841	22.3	1.54	14.2
CNN-LSTM [1]	2814	22.6	1.61	16.1	2763	25.7	1.73	17.3
CNN-XGBoost [38]	3046	23.1	1.82	20.7	3145	24.6	2.07	18.2
CNN-BiLSTM [39]	2863	21.9	1.91	19.1	2985	23.4	2.19	21.9
kNN–GNN [40]	2963	22.4	2.06	20.6	3056	24.1	2.21	22.1
URP-CNN [41]	2814	23.7	2.06	21.1	3048	25.0	2.11	20.6
GAN-SynthRisk [48]	3264	27.9	2.55	25.5	3511	28.7	2.64	26.4
TabTransformer-WLoss [49]	3070	25.4	2.45	24.5	3101	26.1	2.50	25.0
TabTransformer-LIME [50]	3267	30.2	2.42	26.1	3399	31.9	2.61	24.2
GAN-Oversample [51]	3336	30.1	2.84	28.4	3406	31.5	2.93	29.3
TCN-DilateFormer [54]	3211	29.6	2.40	24.0	3321	30.3	2.67	26.7
DAMEL [55]	3637	29.3	2.96	29.6	3708	30.6	2.82	28.2
Proposed	2625	21.6	1.71	18.2	2756	22.9	1.82	17.1

**Fig 4:**
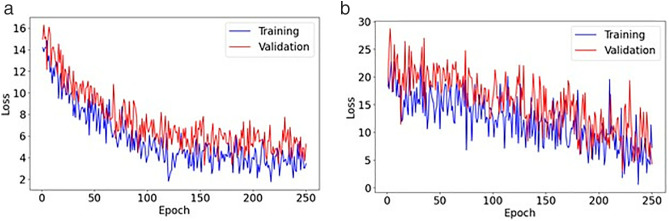
Training and validation loss curves for the proposed model on the a) CSMAR an b) MorningStar datasets in 250 epochs.

Fig 4 shows the training and validation loss trends over 256 epochs for data from the CSMAR and MorningStar datasets. These trends highlight the ability of the model to generalize effectively. In the CSMAR dataset graph, the training and validation losses decrease steadily, aligning closely by the end of training, indicating effective learning without overfitting. The close convergence of these losses demonstrates the generalization of the model, applying learned patterns to both seen and unseen data. Conversely, the MorningStar dataset graph exhibits more fluctuation but similarly shows validation losses closely tracking training losses, indicating robustness against overfitting. These consistent patterns across datasets validate the reliability and stability of the model for complex tasks, such as credit risk prediction, which is essential for practical applications where generalization is crucial for accuracy and reliability in diverse settings.

**Fig 5:**
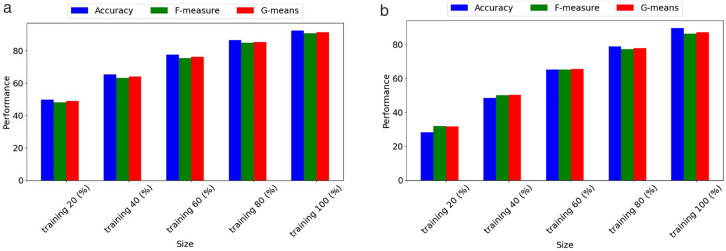
Scalability analysis of the proposed model on the a) CSMAR an b) MorningStar datasets.

Fig 5 illustrates the scalability of our proposed model, demonstrating its performance enhancement as the volume of training data increases from 20% to 100% of the dataset. This analysis was conducted using both the CSMAR and MorningStar datasets. The results indicate that increasing data volumes significantly enhances the accuracy, F-measure, and G-means of the model. This capability is crucial for applications in credit risk prediction, where data availability can be inconsistent. The ability of the model to adapt to increasing data volumes without losing performance effectiveness is especially beneficial in the financial sector, where data volume can fluctuate widely. Furthermore, the robust performance of the model across various data sizes underscores the success of its feature selection and imbalanced classification methods, which are supported by off-policy PPO and precise hyperparameter adjustments via the BOHB algorithm. These methods ensure that the model excels with limited data and scales effectively with larger datasets, demonstrating its readiness for both current and future challenges in diverse financial settings.

**Fig 6:**
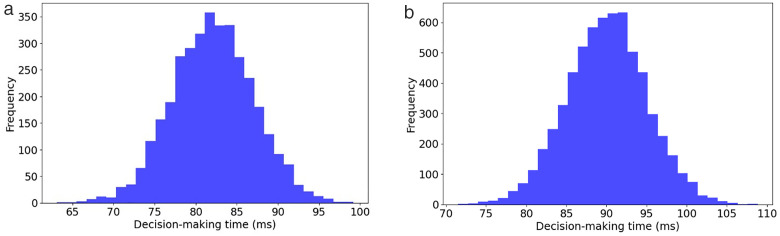
Decision-making time distribution of the proposed model on the a) CSMAR and b) MorningStar datasets.

Fig 6 illustrates the decision-making time distributions for the proposed model when applied to the CSMAR and MorningStar datasets, highlighting its suitability for real-time applications. The histograms indicate that most decision times fall within an optimal and narrow range, demonstrating the rapid processing ability of the model, which is critical for real-time financial market decisions. Notably, decision times peak around 85 milliseconds for CSMAR and 90 milliseconds for MorningStar, indicating consistent and swift performance of the model across various datasets. This consistency is essential for real-world applications where financial decisions have significant impacts, requiring a reliable and fast-performing model. The slight differences in peak times between datasets may reflect variations in data complexity or volume, yet the high processing speed of the model is maintained. Integrating an off-policy PPO for dynamic feature selection and the BOHB algorithm for efficient hyperparameter tuning enhances both learning effectiveness and computational speed, ensuring the model delivers quick and accurate predictions that meet the fast-paced demands of modern financial operations.

#### 4.3.1 Analysis of generalizability.

To confirm the wide applicability of our credit risk prediction model, we carried out detailed tests using the KMV default, GMSC, and UCICCD datasets. The results are shown in Tables 6–8 for the KMV default, GMSC, and UCICCD datasets.

**Table 6:** Performance comparison of the proposed model against advanced studies using the KMV default dataset.

Model	Accuracy	F-measure	G-means	AUC
XGBoost [11]	66.966 ± 0.043	67.399 ± 0.029	68.251 ± 0.063	0.592 ± 0.040
PSO-XGBoost [6]	67.296 ± 0.038	68.081 ± 0.025	68.898 ± 0.059	0.605 ± 0.052
SW-SVDD [27]	68.388 ± 0.074	68.977 ± 0.036	69.812 ± 0.089	0.628 ± 0.087
LightGBM-PCA-SMOTEENN [28]	69.029 ± 0.006	69.565 ± 0.088	70.391 ± 0.096	0.641 ± 0.092
LightGBM [31]	67.720 ± 0.004	68.467 ± 0.025	69.272 ± 0.073	0.622 ± 0.077
TSC-SVM [32]	70.812 ± 0.061	71.203 ± 0.013	71.650 ± 0.058	0.648 ± 0.066
ES-LqSVM [34]	72.315 ± 0.006	72.635 ± 0.083	73.475 ± 0.099	0.674 ± 0.054
SMPSO-NSGA-FS [36]	73.336 ± 0.072	73.967 ± 0.013	74.212 ± 0.069	0.688 ± 0.065
HDNN [37]	75.327 ± 0.005	76.104 ± 0.034	76.981 ± 0.000	0.718 ± 0.081
CNN-LSTM [1]	75.986 ± 0.036	76.663 ± 0.046	77.527 ± 0.083	0.731 ± 0.077
CNN-XGBoost [38]	76.789 ± 0.003	77.231 ± 0.000	78.124 ± 0.008	0.738 ± 0.086
CNN-BiLSTM [39]	77.681 ± 0.035	77.984 ± 0.026	78.851 ± 0.008	0.750 ± 0.025
kNN–GNN [40]	78.148 ± 0.098	78.673 ± 0.009	79.537 ± 0.068	0.764 ± 0.027
URP-CNN [41]	78.527 ± 0.027	79.579 ± 0.092	80.441 ± 0.021	0.775 ± 0.008
GAN-SynthRisk [48]	79.084 ± 0.100	79.870 ± 0.099	80.718 ± 0.031	0.783 ± 0.078
TabTransformer-WLoss [49]	79.487 ± 0.098	80.550 ± 0.026	81.420 ± 0.010	0.786 ± 0.070
TabTransformer-LIME [50]	80.120 ± 0.078	81.200 ± 0.018	82.036 ± 0.031	0.788 ± 0.042
GAN-Oversample [51]	80.499 ± 0.043	81.886 ± 0.065	82.722 ± 0.001	0.792 ± 0.045
TCN-DilateFormer [54]	81.379 ± 0.049	82.634 ± 0.004	83.448 ± 0.038	0.798 ± 0.064
DAMEL [55]	81.982 ± 0.046	83.027 ± 0.049	83.835 ± 0.048	0.801 ± 0.028
Proposed	85.682 ± 0.032	87.047 ± 0.037	88.589 ± 0.087	0.814 ± 0.070

**Table 7:** Performance comparison of the proposed model against advanced studies using the GMSC dataset.

Model	Accuracy	F-measure	G-means	AUC
XGBoost [11]	69.812 ± 0.005	70.803 ± 0.019	71.650 ± 0.043	0.599 ± 0.024
PSO-XGBoost [6]	70.695 ± 0.006	71.523 ± 0.074	72.389 ± 0.044	0.611 ± 0.096
SW-SVDD [27]	70.991 ± 0.077	72.443 ± 0.060	73.285 ± 0.031	0.624 ± 0.026
LightGBM-PCA-SMOTEENN [28]	72.871 ± 0.009	73.125 ± 0.041	73.945 ± 0.036	0.633 ± 0.024
LightGBM [31]	69.634 ± 0.028	70.738 ± 0.012	72.522 ± 0.007	0.621 ± 0.027
TSC-SVM [32]	73.935 ± 0.045	74.263 ± 0.067	75.022 ± 0.083	0.665 ± 0.043
ES-LqSVM [34]	74.836 ± 0.005	74.930 ± 0.087	75.696 ± 0.080	0.674 ± 0.048
SMPSO-NSGA-FS [36]	76.571 ± 0.020	76.520 ± 0.099	77.304 ± 0.050	0.683 ± 0.092
HDNN [37]	78.141 ± 0.079	79.397 ± 0.094	80.164 ± 0.036	0.700 ± 0.008
CNN-LSTM [1]	79.579 ± 0.006	80.567 ± 0.073	81.720 ± 0.043	0.713 ± 0.011
CNN-XGBoost [38]	79.834 ± 0.033	80.857 ± 0.062	80.906 ± 0.058	0.719 ± 0.090
CNN-BiLSTM [39]	80.217 ± 0.017	81.548 ± 0.009	82.319 ± 0.019	0.734 ± 0.058
kNN–GNN [40]	80.612 ± 0.065	82.132 ± 0.021	83.277 ± 0.039	0.741 ± 0.054
URP-CNN [41]	81.946 ± 0.030	82.591 ± 0.054	83.319 ± 0.049	0.754 ± 0.074
GAN-SynthRisk [48]	82.747 ± 0.067	83.229 ± 0.039	83.937 ± 0.064	0.766 ± 0.081
TabTransformer-WLoss [49]	83.096 ± 0.042	84.668 ± 0.095	85.373 ± 0.072	0.778 ± 0.096
TabTransformer-LIME [50]	83.439 ± 0.090	84.334 ± 0.071	85.603 ± 0.023	0.785 ± 0.081
GAN-Oversample [51]	83.970 ± 0.035	85.250 ± 0.032	86.531 ± 0.062	0.800 ± 0.017
TCN-DilateFormer [54]	84.769 ± 0.089	85.620 ± 0.021	86.604 ± 0.049	0.814 ± 0.040
DAMEL [55]	85.727 ± 0.010	86.885 ± 0.044	87.589 ± 0.024	0.818 ± 0.093
Proposed	89.240 ± 0.022	90.576 ± 0.046	90.888 ± 0.024	0.831 ± 0.088

**Table 8:** Performance comparison of the proposed model against advanced studies using the UCICCD dataset.

Model	Accuracy	F-measure	G-means	AUC
XGBoost [11]	68.812 ± 0.091	69.803 ± 0.066	71.650 ± 0.029	0.596 ± 0.066
PSO-XGBoost [6]	69.309 ± 0.062	70.354 ± 0.014	71.798 ± 0.066	0.606 ± 0.091
SW-SVDD [27]	70.268 ± 0.085	71.681 ± 0.099	72.528 ± 0.007	0.622 ± 0.080
LightGBM-PCA-SMOTEENN [28]	71.127 ± 0.005	72.352 ± 0.024	73.217 ± 0.079	0.630 ± 0.065
LightGBM [31]	68.046 ± 0.064	70.847 ± 0.037	71.688 ± 0.059	0.619 ± 0.033
TSC-SVM [32]	71.372 ± 0.095	72.511 ± 0.034	74.372 ± 0.099	0.652 ± 0.022
ES-LqSVM [34]	73.872 ± 0.060	74.203 ± 0.040	75.031 ± 0.056	0.669 ± 0.005
SMPSO-NSGA-FS [36]	75.600 ± 0.001	76.673 ± 0.023	77.499 ± 0.025	0.676 ± 0.028
HDNN [37]	76.272 ± 0.071	76.288 ± 0.077	77.089 ± 0.039	0.691 ± 0.027
CNN-LSTM [1]	78.956 ± 0.058	79.028 ± 0.043	80.799 ± 0.056	0.703 ± 0.065
CNN-XGBoost [38]	79.631 ± 0.036	80.536 ± 0.062	81.283 ± 0.061	0.710 ± 0.090
CNN-BiLSTM [39]	79.315 ± 0.029	80.245 ± 0.091	82.975 ± 0.077	0.727 ± 0.095
kNN–GNN [40]	80.910 ± 0.016	81.863 ± 0.076	82.614 ± 0.063	0.731 ± 0.054
URP-CNN [41]	80.637 ± 0.004	82.566 ± 0.036	83.327 ± 0.002	0.745 ± 0.017
GAN-SynthRisk [48]	81.500 ± 0.065	82.063 ± 0.004	83.799 ± 0.067	0.759 ± 0.019
TabTransformer-WLoss [49]	82.162 ± 0.042	83.628 ± 0.005	84.352 ± 0.094	0.769 ± 0.032
TabTransformer-LIME [50]	82.495 ± 0.067	83.032 ± 0.096	84.756 ± 0.052	0.790 ± 0.087
GAN-Oversample [51]	82.782 ± 0.004	83.512 ± 0.096	84.250 ± 0.093	0.804 ± 0.077
TCN-DilateFormer [54]	83.568 ± 0.091	84.226 ± 0.045	85.982 ± 0.083	0.813 ± 0.056
DAMEL [55]	84.331 ± 0.027	85.927 ± 0.026	86.681 ± 0.094	0.815 ± 0.057
Proposed	88.075 ± 0.027	89.485 ± 0.040	90.269 ± 0.030	0.821 ± 0.043

Testing our model on the KMV database demonstrated substantial enhancements across all principal performance metrics, highlighting its effective adaptability across various financial settings. Several recent models, including TSC-SVM, ES-LqSVM, and SMPSO-NSGA-FS, have shown better results than older machine-learning approaches. However, they still performed worse than deep learning-based strategies. Deep learning models such as GAN-SynthRisk, TabTransformer-WLoss, TabTransformer-LIME, GAN-Oversample, and TCN-DilateFormer delivered significant performance improvements. Among them, DAMEL achieved the best results with an accuracy of 81.982% and an AUC of 0.801. Although DAMEL achieved the strongest performance among prior models, the proposed model surpassed it with an accuracy of 85.682%, marking an improvement of 3.7 percentage points. It also showed superior F-measure and G-means scores, 87.047% and 88.589%, respectively, exceeding DAMEL by 4.02 and 4.75 percentage points. Additionally, it achieved an AUC of 0.814, surpassing DAMEL by 0.013 points.

Consistent improvements were also observed in the GMSC dataset. While prior models, such as CNN-BiLSTM, kNN–GNN, and GAN-SynthRisk, showed notable performance, DAMEL again delivered the best prior result, with an accuracy of 85.727% and an AUC of 0.818. However, our model exceeded these benchmarks. It achieved an accuracy of 89.240%, an F-measure of 90.576%, a G-means of 90.888%, and an AUC of 0.831.

A similar trend emerged when testing on the UCICCD dataset. The proposed model achieved an accuracy of 88.075%, an F-measure of 89.485%, a G-means of 90.269%, and an AUC of 0.821. These results surpass those of DAMEL. That model previously achieved the top performance, with an accuracy of 84.331% and an AUC of 0.815.

The performance gains across all three datasets are attributed to the comprehensive integration of feature selection, imbalanced classification management, and meticulous hyperparameter optimization in our model. The off-policy PPO algorithm, applied for feature selection, accurately identifies essential features that significantly enhance predictive accuracy by focusing on key credit risk indicators. This technique also ensures adequate representation of minority classes, improving the ability of the model to generalize across diverse and uneven datasets. Furthermore, using BOHB for hyperparameter tuning optimizes model parameters across various datasets and conditions, accelerating the optimization process while identifying the best configurations for maximum robustness and generalizability.

#### 4.3.2 Analysis of off-policy PPO.

**Fig 7:**
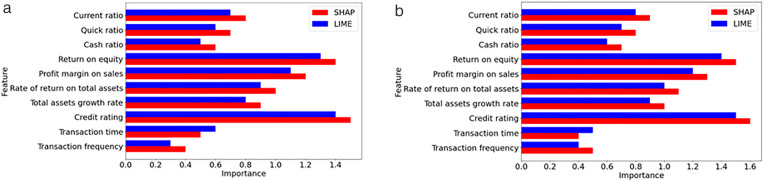
Comparative analysis of feature importance using the SHAP and LIME explanations on the a) CSMAR and b) MorningStar datasets.

To enhance interpretability and verify the effectiveness of our feature selection process, we employed SHAP and LIME. Fig 7 illustrates the importance of various features in predicting credit risk across the CSMAR and MorningStar datasets. Examining the SHAP and LIME values provides clear insights into the impact of various features on credit risk predictions, revealing unique patterns in the two datasets. Key financial metrics, such as “Return on Equity,” “Profit Margin on Sales,” and “Credit Rating,” consistently demonstrate high importance, underscoring their critical role in financial analysis. “Return on Equity” indicates the profitability of a company compared to the equity of its shareholders, marking it as an essential measure of financial health and efficiency. Similarly, “Profit Margin on Sales” reflects operational efficiency by showing how sales translate into profits. “Credit Rating” is crucial for risk assessment, with higher ratings indicating lower risk. Liquidity measures like “Current Ratio,” “Quick Ratio,” and “Cash Ratio” are also significant and crucial for assessing the ability of a company to meet short-term financial obligations. The ability of SHAP and LIME to highlight these features demonstrates the advantages of using the off-policy PPO approach in our feature selection process. This method boosts model performance by focusing on relevant data and improves interpretability by aligning feature importance with fundamental financial principles. The use of SHAP and LIME explanations, along with off-policy PPO, has confirmed that selecting key financial indicators is critical for predicting credit risk, thereby enhancing both the robustness of the model and the clarity of the results. This enables stakeholders to make informed decisions based on reliable and interpretable data.

Table 9 presents the performance of the proposed model on majority and minority classes within the CSMAR and MorningStar datasets, showcasing the effectiveness of off-policy PPO in addressing class imbalance in classification tasks. The analysis indicates that the model secures impressive accuracy, F-measure, G-means, and AUC scores across both datasets. This performance is notable given the challenges posed by imbalanced data, where minority classes often have fewer examples and are more difficult to predict accurately. The critical role of off-policy PPO in managing class imbalance becomes apparent upon a detailed examination of the performance metrics. Off-policy PPO allows the model to efficiently incorporate lessons from past experiences into its learning process rather than limiting it to data from current interactions alone. This functionality is advantageous in environments with imbalanced datasets because it enables the model to leverage historical data that may be underrepresented in current samples. It also enhances its ability to generalize effectively across both majority and minority classes.

**Table 9:** Performance evaluation of the proposed model on majority and minority classes for the CSMAR and MorningStar datasets.

Metric	Dataset			
	CSMAR		MorningStar	
	Majority	Minority	Majority	Minority
Accuracy	94.478 ± 0.075	90.478 ± 0.085	91.692 ± 0.064	87.692 ± 0.068
F-measure	93.763 ± 0.076	87.763 ± 0.082	88.358 ± 0.074	84.358 ± 0.079
G-means	94.378 ± 0.016	88.378 ± 0.018	89.123 ± 0.076	85.123 ± 0.072
AUC	0.909 ± 0.005	0.869 ± 0.007	0.883 ± 0.085	0.843 ± 0.088

For the CSMAR dataset, despite the expected differences between the majority and minority classes, the model shows a less than 4% difference in accuracy and approximately a 6% variance in F-measure. In the MorningStar dataset, although the disparities are slightly larger, the model still maintains strong performance, demonstrating robustness in challenging conditions that usually amplify the problems associated with class imbalance. The G-means and AUC metrics further validate the ability of the model to effectively balance sensitivity and specificity, which is vital in credit risk evaluation where failing to identify a high-risk account could have substantial financial consequences. The integration of off-policy PPO not only boosts the accuracy and generalization capabilities of the model but also ensures its resilience across various operational conditions. Such robustness is essential for real-world financial applications, where unpredictable class distributions could result in biased predictions and inaccurate risk assessments. Therefore, implementing off-policy PPO substantially reduces the usual predictive bias towards the majority class, facilitating more fair and reliable risk evaluations across all classes. This method enhances the predictive accuracy of the model and increases the reliability of its outputs, which is crucial for stakeholders relying on these predictions to make informed financial decisions.

**Fig 8:**
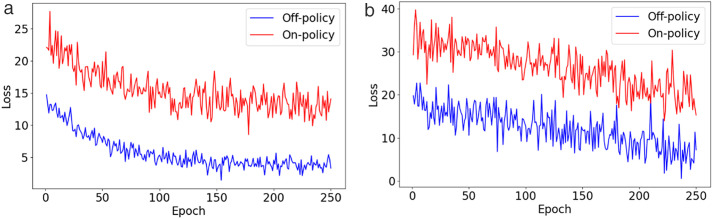
Comparative analysis of off-policy PPO algorithm and on-policy algorithm on the a) CSMAR and b) MorningStar datasets.

To illustrate the superior performance of off-policy PPO in our model, Fig 8 displays the loss trends over 250 iterations for off-policy PPO compared to the traditional on-policy algorithm across the CSMAR and MorningStar datasets. The graphs show that the off-policy PPO decreases loss more smoothly and more rapidly than the on-policy method. In particular, for the CSMAR dataset, the off-policy PPO quickly reduces loss early in training and maintains a lower loss level, unlike the on-policy PPO, which shows greater loss fluctuations and a slower reduction. This suggests that off-policy PPO more effectively utilizes historical data, thereby enhancing generalization and preventing overfitting. The MorningStar dataset shows similar trends, with the off-policy PPO starting at lower losses and maintaining a steady decline, achieving better stability throughout training. In contrast, the on-policy PPO exhibits higher loss and more pronounced fluctuations, suggesting less effective learning and adaptation to changes in data. The ability of off-policy PPO to draw from a wider range of past interactions allows it to generalize across diverse financial scenarios more effectively than on-policy methods, which rely mainly on recent data. This broad learning scope enhances the adaptability of off-policy PPO. It reduces its susceptibility to variance and overfitting, making it more reliable for complex financial modeling and ensuring improved predictive accuracy and training dynamics.

#### 4.3.3 Analysis of BOHB.

We aim to compare the implementation of BOHB against a range of established hyperparameter optimization techniques. To ensure fair assessment, uniformity was maintained across all model variables during evaluations. The analysis encompasses three basic algorithms—random search, grid search, and Bayesian optimization—and six evolutionary algorithms, including the salp swarm algorithm (SSA), human mental search (HMS), bat algorithm (BA), firefly algorithm (FA), artificial bee colony (ABC), and differential evolution (DE). We evaluated the performance of these algorithms using the CSMAR and MorningStar datasets, detailed in Tables 10 and 11.

**Table 10:** Performance metrics comparison of basic and metaheuristic techniques for hyperparameter optimization across the CSMAR dataset.

Algorithm	Accuracy	F-measure	G-means	AUC
Random search	76.710 ± 0.015	77.351 ± 0.096	77.966 ± 0.092	0.745 ± 0.098
Grid search	77.100 ± 0.062	77.986 ± 0.005	78.597 ± 0.053	0.753 ± 0.099
Bayesian optimization	84.817 ± 0.087	85.592 ± 0.014	86.070 ± 0.090	0.855 ± 0.079
Hyperband	83.442 ± 0.052	84.462 ± 0.054	84.953 ± 0.056	0.839 ± 0.021
SSA	79.917 ± 0.030	79.716 ± 0.057	80.286 ± 0.030	0.766 ± 0.070
HMS	80.439 ± 0.033	80.619 ± 0.014	81.178 ± 0.016	0.772 ± 0.026
BA	81.808 ± 0.009	81.464 ± 0.018	82.026 ± 0.052	0.790 ± 0.049
FA	81.138 ± 0.042	82.218 ± 0.012	82.769 ± 0.025	0.804 ± 0.054
ABC	82.518 ± 0.002	82.895 ± 0.095	83.418 ± 0.010	0.814 ± 0.096
DE	81.914 ± 0.058	83.702 ± 0.080	84.215 ± 0.057	0.832 ± 0.100
BOHB	92.478 ± 0.075	90.763 ± 0.076	91.378 ± 0.016	0.889 ± 0.005

**Table 11:** Performance metrics comparison of basic and metaheuristic techniques for hyperparameter optimization across the MorningStar dataset.

Algorithm	Accuracy	F-measure	G-means	AUC
Random search	75.113 ± 0.093	75.811 ± 0.015	76.447 ± 0.010	0.649 ± 0.097
Grid search	75.410 ± 0.080	76.716 ± 0.038	77.348 ± 0.072	0.661 ± 0.091
Bayesian optimization	82.003 ± 0.079	83.488 ± 0.054	84.058 ± 0.081	0.821 ± 0.095
Hyperband	82.682 ± 0.032	84.047 ± 0.037	84.589 ± 0.087	0.838 ± 0.095
SSA	76.167 ± 0.010	77.586 ± 0.088	78.202 ± 0.048	0.670 ± 0.045
HMS	76.843 ± 0.052	78.245 ± 0.014	78.849 ± 0.010	0.682 ± 0.082
BA	77.762 ± 0.037	79.047 ± 0.068	79.626 ± 0.100	0.698 ± 0.077
FA	78.715 ± 0.048	79.725 ± 0.090	80.271 ± 0.022	0.708 ± 0.096
ABC	79.590 ± 0.031	80.390 ± 0.056	80.934 ± 0.036	0.722 ± 0.018
DE	79.912 ± 0.015	81.033 ± 0.003	81.591 ± 0.069	0.734 ± 0.055
BOHB	89.692 ± 0.064	86.358 ± 0.074	87.123 ± 0.076	0.863 ± 0.085

For the CSMAR dataset, the results clearly show that the BOHB algorithm outperforms traditional and evolutionary algorithms. The metrics indicate that BOHB not only leads in every performance category but also achieves these leads by considerable margins, highlighting its capability to manage complex optimization challenges effectively. Focusing on the improvement rates, the accuracy of BOHB is particularly impressive. It achieves an accuracy of 92.478%, which is approximately 7.661% higher than the next best method, Bayesian optimization, with a score of 84.817%. This gap highlights the strength of BOHB in determining the optimal model parameters, which notably increases predictive accuracy. In the F-measure category, BOHB achieves 90.763%, surpassing the highest F-measure recorded by any evolutionary algorithm, with differential evolution at 83.702%, a boost of roughly 7.061%. This indicates the efficiency of BOHB in balancing precision and recall, a critical factor in handling datasets with class imbalances typical in financial models. In G-means, which evaluates the balance between sensitivity and specificity, BOHB scores 91.378%, surpassing the 84.215% achieved by differential evolution by 7.163%. This improvement shows that BOHB maintains consistent model performance across various classes, which is crucial for fair and unbiased risk assessments. Lastly, in the AUC metric, which measures the ability of the model to distinguish between classes, BOHB scores 0.889, outshining the top score from other methods, where differential evolution achieves an AUC of 0.832. This increase of 5.7% signals a significant boost in the capability of the model to accurately differentiate between levels of risk, which is essential for precise credit risk prediction.

For the MorningStar dataset, the results demonstrate the superior effectiveness of the BOHB approach over basic and metaheuristic optimization techniques. Among the basic optimization methods, Bayesian optimization and Hyperband exhibit strong performance. However, when examining metaheuristic algorithms such as the SSA, HMS, BA, FA, ABC, and DE, it is evident that there is a noticeable increase in performance as algorithms grow in complexity. BOHB outperforms all other methods, achieving an accuracy rate of 89.692%, which is approximately 7% higher than Hyperband, the next highest performer, with a score of 82.682%. This substantial margin underscores the exceptional ability of BOHB to navigate hyperparameter spaces more effectively and precisely. Regarding the F-measure, BOHB achieves 86.358%, outdoing Bayesian optimization, which is the top performer among the basic methods, with a score of 83.488%. This improvement is vital as it shows the strength of BOHB in balancing precision and recall, which is crucial for classification models, especially in a diverse dataset like MorningStar. G-means, which assesses the balance between sensitivity and specificity, also places BOHB at the forefront with a score of 87.123%. This score is significantly higher than the 84.589% achieved by Hyperband, reflecting an improvement of approximately 3%. This metric highlights the ability of BOHB to maintain consistent performance across different classes, which enhances the reliability and fairness of the model in risk prediction. The AUC results further reinforce the dominance of BOHB, achieving a score of 0.863, which is notably higher than the 0.838 obtained by Hyperband. This top score suggests a more effective capability of BOHB to discriminate between classes, which is essential for accurate risk stratification in financial datasets.

The statistical analysis confirms the superiority of the BOHB algorithm over other hyperparameter optimization methods for the CSMAR and MorningStar datasets, as indicated by p-values that are significantly below the standard threshold of 0.05. For the CSMAR dataset, the BOHB algorithm achieves remarkable improvements in accuracy, F-measure, G-means, and AUC, scoring 92.478, 90.763, 91.378, and 0.889, respectively. These results represent statistically significant enhancements compared to the next best-performing algorithm, DE, which scored 81.914, 83.702, 84.215, and 0.832. These findings translate to an improvement rate of approximately 12.9% in accuracy and 8.5% in AUC. Similarly, in the MorningStar dataset, the BOHB algorithm maintains superior performance, achieving an accuracy of 89.692 and an F-measure of 86.358 compared to the scores of 82.682 and 84.047 achieved by Hyperband. These improvements correspond to an 8.5% increase in accuracy and a 5.5% enhancement in F-measure, with p-values supporting the statistical significance of these differences. All confidence intervals were calculated at the 95% level based on the differences in paired sample means. The calculation used a t-distribution and assumed that the performance differences across cross-validation folds followed an approximately normal distribution. The improvements are consistent across all metrics and datasets, with narrow confidence intervals that surpass the metrics of competing algorithms. This analysis confirms the ability of the BOHB algorithm to effectively optimize hyperparameters, ensuring reliable performance across various datasets and metrics. The strong statistical evidence underscores its reliability for hyperparameter tuning in critical applications such as credit risk assessment.

Table 12 comprehensively evaluates the computational efficiency of various hyperparameter optimization algorithms for the CSMAR and MorningStar datasets, comparing runtime, GPU memory usage, and FLOPs. Among the tested methods, BOHB achieves a strong balance between resource usage and performance. BOHB records a runtime of 2625 seconds on CSMAR and 2756 seconds on MorningStar. These values are significantly lower than those of computationally intensive methods such as BA, ABC, and DE, which range between 3245 and 3696 seconds. In terms of FLOP, BOHB consumes only 1.71 × 10¹⁰ and 1.82 × 10¹⁰ operations on CSMAR and MorningStar, respectively. This performance surpasses that of other metaheuristic algorithms, such as FA, SSA, and HMS, all of which exceed 2.4 × 10¹⁰ FLOPs. While simpler techniques, such as random search and Hyperband, show slightly lower FLOPs, they often lack the precision and convergence reliability required in complex, high-dimensional search spaces. BOHB integrates Bayesian optimization and Hyperband to efficiently explore the parameter space. This integration helps reduce unnecessary computation, making BOHB a strong option for scalable and efficient hyperparameter tuning in financial prediction tasks.

**Table 12:** Comparative analysis of computational efficiency across various hyperparameter optimization algorithms on the CSMAR and MorningStar datasets.

Algorithm	Dataset					
	CSMAR			MorningStar		
	Runtime	GPU	FLOP (${\times \text{10}}^{\text{10}}$)	Runtime	GPU	FLOP ($\times {\text{10}}^{\text{10}}$)
Random search	2169	18.5	1.62	2274	20.4	1.71
Grid search	3074	22.7	1.96	3165	23.1	2.04
Bayesian optimization	2945	23.8	2.15	3058	25.9	2.24
Hyperband	2415	20.2	1.54	2539	22.4	1.68
SSA	2980	23.6	2.34	3045	24.6	2.46
HMS	2996	24.1	2.52	2869	26.3	2.61
BA	3245	25.6	2.74	3385	26.7	2.84
FA	3342	24.1	2.63	3418	26.3	2.71
ABC	3415	26.5	2.81	3696	27.1	2.92
DE	3352	23.1	2.42	3415	24.6	2.59
BOHB	2625	21.6	1.71	2756	22.9	1.82

**Fig 9:**
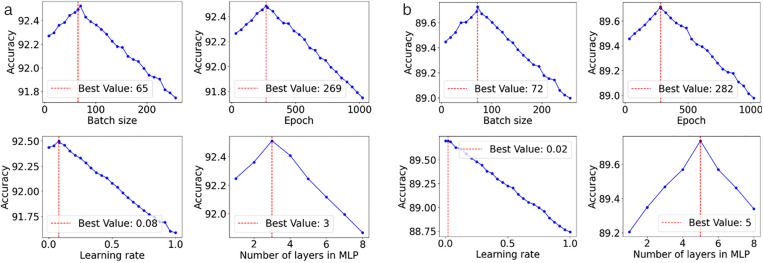
Hyperparameter optimization analysis across the a) CSMAR and b) MorningStar datasets.

Fig 9 provides an in-depth analysis of the effectiveness of the BOHB algorithm in optimizing hyperparameters for improved model performance. BOHB adjusts key parameters, including batch size, number of epochs, learning rate, and MLP layers, to identify configurations that maximize accuracy in credit risk prediction models for the CSMAR and MorningStar datasets. For batch size, optimal values are 65 for CSMAR and 72 for MorningStar, striking a balance between computational efficiency and learning quality. Epoch analysis reveals that training for 269 on CSMAR and 282 on MorningStar achieves the best results, avoiding overfitting and demonstrating the ability of BOHB to tailor stopping points to dataset characteristics. The learning rate findings emphasize the importance of precise tuning to prevent slow convergence or loss function overshooting, with optimal rates identified as 0.08 for CSMAR and 0.02 for MorningStar. These results indicate that each dataset benefits from distinct learning rates to minimize errors effectively. Furthermore, the optimal number of MLP layers is three for CSMAR and five for MorningStar, suggesting that MorningStar requires a deeper network to capture its more intricate data patterns. This accurate hyperparameter adjustment illustrates the capability of BOHB to efficiently navigate complex hyperparameter spaces. By combining Bayesian optimization and Hyperband, BOHB enhances model accuracy and ensures robust generalization on unseen data. This hybrid approach minimizes the risks of underfitting and overfitting, thereby confirming its value in enhancing predictive performance across various datasets.

#### 4.3.4 Discussion.

This article introduces an advanced credit risk prediction model tailored for publicly traded companies, addressing challenges in feature selection, imbalanced classification, and hyperparameter optimization. The model integrates off-policy PPO for feature selection, imbalanced classification, and the BOHB for efficient hyperparameter tuning. These components function collectively to enhance the predictive accuracy of the model and ensure consistent performance across various datasets. Experiments demonstrated that our model outperforms existing advanced technologies, with ablation studies revealing the individual contributions of each component.

Table 13 presents a detailed comparative analysis of the proposed model with the best-performing existing methods across five benchmark datasets: CSMAR, MorningStar, KMV, GMSC, and UCICCD. The proposed model consistently outperforms all previous models in every performance metric. On the CSMAR dataset, it achieves a 4.906% increase in accuracy and a 3.125% gain in AUC compared to DAMEL and kNN–GNN, respectively. Similarly, on MorningStar, improvements of 4.252% in accuracy and 2.3% in AUC are observed over DAMEL. Notably, the KMV dataset exhibits substantial advancements, with a 4.02% gain in F-measure and a 4.754% increase in G-means. Comparable improvements are evident in the two public datasets. On the GMSC dataset, the proposed model achieves an accuracy improvement of 3.513%. It also achieves notable gains of 3.691% in F-measure, 3.299% in G-means, and 1.3% in AUC. These results surpass those of DAMEL, the previous top-performing model. The UCICCD dataset shows a similar pattern, with accuracy improving by 3.744%, F-measure by 3.558%, and G-means by 3.588%. The AUC also increased by 0.6%, indicating a modest but consistent improvement. These consistent improvements across different datasets and evaluation metrics highlight the robustness, adaptability, and generalizability of the proposed model, particularly in real-world financial risk prediction scenarios.

**Table 13:** Comparative performance of the proposed model vs. best-existing models on CSMAR, MorningStar, KMV, GMSC, and UCICCD datasets.

Dataset		Accuracy	F-measure	G-means	AUC
CSMAR	Best previous model	DAMEL (87.572%)	DAMEL (87.826%)	DAMEL (88.831%)	kNN–GNN (0.832)
	Performance of the proposed model	92.478%	90.763%	91.378%	0.889
	Percentage difference	+4.906%	+2.937%	+2.547%	+3.125%
MorningStar	Best previous model	DAMEL (85.440%)	GAN-Oversample (84.618%)	TCN-DilateFormer (85.961%)	DAMEL (0.840)
	Performance of the proposed model	89.692%	86.358%	87.123%	0.863
	Percentage difference	+4.252%	+2.086%	+1.162%	+2.3%
KMV	Best previous model	DAMEL (81.982%)	DAMEL (83.027%)	DAMEL (83.835%)	DAMEL (0.801)
	Performance of the proposed model	85.682%	87.047%	88.589%	0.814
	Percentage difference	+3.7%	+4.02%	+4.754%	+1.3%
GMSC	Best previous model	DAMEL (85.727%)	DAMEL (86.885%)	DAMEL (87.589%)	DAMEL (0.818)
	Performance of the proposed model	89.240%	90.576%	90.888%	0.831
	Percentage difference	+3.513%	+3.691%	+3.299%	+1.3%
UCICCD	Best previous model	DAMEL (84.331%)	DAMEL (85.927%)	DAMEL (86.681%)	DAMEL (0.815)
	Performance of the proposed model	88.075%	89.485%	90.269%	0.821
	Percentage difference	+3.744%	+3.558%	+3.588%	+0.6%

This strong performance stems from the integration of two methodologically grounded components: off-policy PPO and BOHB. Off-policy PPO is used for feature selection and class imbalance handling. It utilizes historical policy data to identify key features and learn from underrepresented classes, which is crucial in datasets with skewed financial distributions. BOHB is used for tuning hyperparameters efficiently in high-dimensional spaces. It speeds up the search for optimal settings and reduces the need for extensive computation. Together, these components form a theoretically sound and practically scalable pipeline tailored to the structural complexities of credit risk modeling. These components were tested successfully on the CSMAR, MorningStar, KMV, GMSC, and UCICCD datasets. The results confirm that the model is robust and suitable for real-world financial environments.

Beyond technical performance, ethical considerations are vital when developing and deploying credit risk prediction models. This study utilizes a combination of proprietary and publicly available datasets. The CSMAR, MorningStar, and KMV datasets are proprietary financial databases accessible only through licensed institutional agreements. In contrast, the GMSC and UCICCD datasets are publicly available, anonymized, and commonly used for academic research. All datasets used in this study are de-identified and comply with standard data privacy regulations. The model is evaluated on historical data. However, applying it in real-world financial systems, such as loan approvals or insurance decisions, raises important ethical concerns. If used without oversight, such models may lead to algorithmic bias and unfair treatment of underrepresented or economically vulnerable groups. To mitigate such risks, the proposed model is intended strictly for academic and analytical evaluation. Any use of similar models in operational settings must ensure transparency, fairness, and regular auditing. Ethical deployment entails striking a balance between predictive accuracy and social accountability, legal standards, and protections against unintended consequences at every stage of the model’s use. These findings are intended to support methodological advancement rather than serving as direct decision-making tools. Applying these models in practice requires additional validation, domain-specific adjustments, and adherence to regulatory compliance.

The design and capabilities of the proposed model make it highly applicable to domains beyond credit risk prediction, such as fraud detection, healthcare, and retail analytics. These areas face similar challenges, including imbalanced data distributions, high-dimensional features, and the need for accurate decisions under uncertainty. To test the model across other domains, we utilized three publicly available benchmark datasets. These include the NHIS healthcare claims and fraud (NHISHCF) dataset [63] for healthcare fraud detection, the UCI heart disease (UCIHD) dataset [64] for heart disease detection, and the Online Retail II UCI (ORIIUCI) dataset [65] for retail analytics. The experimental results, presented in Table 14, demonstrate that the model achieves strong and consistent performance across all domains. Accuracy exceeds 89%, F-measure remains above 90%, and AUC is higher than 0.82 in each case. These strong results demonstrate that the model can generalize well across domains. It achieves this by addressing challenges such as sparse features, noisy data, and underrepresented classes. The model performs well in multiple sectors, showing its scalability and flexibility. This makes it suitable for a wide range of real-world prediction tasks.

**Table 14:** Comparative performance of the proposed model across NHISHCF, UCIHD, and ORIIUCI.

Dataset	Accuracy	F-measure	G-means	AUC
NHISHCF	89.514 ± 0.022	90.792 ± 0.019	91.541 ± 0.089	0.826 ± 0.042
UCIHD	91.125 ± 0.031	92.387 ± 0.063	92.128 ± 0.091	0.843 ± 0.077
ORIIUCI	90.604 ± 0.006	91.893 ± 0.074	91.614 ± 0.091	0.831 ± 0.047

***Limitations*.** The limitations of the proposed model can be summarized as follows:

- Limited adaptability to market dynamics: The model is trained using static historical datasets, which limits its ability to respond to changing market conditions. These datasets may not capture rapid fluctuations in financial environments that result from macroeconomic shifts, new regulations, or geopolitical events. This mismatch can degrade predictive accuracy over time. To ensure continued accuracy, regular model retraining should be implemented upon detection of concept drift. Additionally, incorporating online learning techniques or adaptive architectures such as transfer learning may help the model update incrementally with new data. Integration with real-time data monitoring systems also enables automatic updates without requiring manual intervention.
- Computational resource demands: The model requires GPU acceleration and substantial memory resources. This dependency creates challenges for deployment in real-time or resource-limited environments, especially across decentralized financial networks. This can be addressed through inference optimization methods such as model pruning, quantization, and knowledge distillation. These approaches significantly reduce computational cost while maintaining performance. Cloud-based deployment with edge computing for caching and local prediction can strike a balance between speed and scalability, providing a more efficient solution.
- Dependence on input data quality: Inaccurate labels, missing values, and corrupted features may reduce predictive accuracy and cause systemic bias. This issue is critical in financial datasets, where minor errors can result in costly consequences. Implementing robust data validation pipelines, including missing data imputation, noise filtering, and outlier detection, can enhance dataset integrity before training. Additionally, incorporating robust learning techniques such as noise-tolerant loss functions and ensemble models improves resilience to data imperfections.
- Manual configuration requirements: Although hyperparameter tuning is automated through BOHB, certain components still require manual setup. These include the structure of the reinforcement learning reward, intervals for policy updates, and preprocessing strategies for features. These dependencies limit scalability and reproducibility. Future work can explore the use of AutoML frameworks to automate model design decisions, reward shaping, and feature engineering. Meta-learning techniques can also be adapted to generalize across datasets, minimizing the need for repeated manual configuration.
- Lack of real-time evaluation: The current evaluation only includes static batch-mode experiments. It does not cover streaming data or real-time processing, which are important in dynamic financial environments that demand rapid risk assessment. To enable real-time applicability, future research should implement the model within stream processing platforms (e.g., Apache Kafka or Apache Flink). This would allow evaluation under real-world latency constraints and facilitate integration with real-time decision support systems.

## 5. Conclusion

The proposed credit risk prediction model represents a significant advancement in addressing the challenges of feature selection, imbalanced classification, and hyperparameter optimization within financial datasets. The model enhances predictive accuracy and operational efficiency by combining the off-policy PPO algorithm with the BOHB method. Experimental evaluations on the CSMAR, MorningStar, KMV, GMSC, and UCICCD datasets demonstrate the superior performance of the model, achieving F-measures of 90.763%, 86.358%, 87.047%, 90.576%, and 89.485%, respectively. These findings reveal improvements over traditional and advanced approaches, highlighting the robustness and adaptability of the model to various financial scenarios. The practical implications of these results are substantial, providing a reliable and efficient tool for credit risk management that supports better financial decision-making for banks, regulatory bodies, and investors.

To make the model more practical, future versions should include real-time data processing. This would support dynamic and ongoing credit risk assessments. This capability is crucial for responding to rapidly changing market conditions, shifts in company credit behavior, and unexpected financial events. Using the model in live banking environments would enable institutions to quickly evaluate credit risks and react more effectively to new challenges. Future research should also explore how explainable artificial intelligence (XAI) techniques can improve model transparency. These methods help stakeholders understand how the model makes decisions, which is especially important in financial situations such as loan approvals. Another useful direction is to expand the validation of the model to other financial fields like insurance, personal lending, and fintech services. Testing the model in international markets with different economic and regulatory systems would help evaluate its flexibility and inform market-specific adjustments. These improvements would create a stronger, clearer, and more adaptable framework for credit risk prediction.

## Supporting information

S1 Appendix Appendix A. (DOCX)
