# Supplementary material for: Credit risk prediction model for listed companies based on improved reinforcement learning and Bayesian optimization hyperband
Source: PLoS One. 2025 Oct 28;20(10):e0332150. doi: 10.1371/journal.pone.0332150 (PMC12561926; doi:10.1371/journal.pone.0332150)
Supplement: S1 Appendix — (DOCX) [file pone.0332150.s001.docx]

**Appendix A:** **Pseudo-code of BOHB**

Algorithm 2 outlines the pseudocode for hyperparameter optimization using BOHB. BOHB explores the hyperparameter space efficiently by integrating the model-based search of BO with the resource allocation strategy of HB. This approach aims to identify high-performing hyperparameter configurations while effectively managing computational resources.

In this algorithm, the primary goal is to optimize hyperparameters, denoted by the variable $x$. Examples of such hyperparameters include learning rate and batch size, among others, which vary depending on the specific machine-learning model being tuned. The algorithm maintains a historical evaluation dataset $D$, which consists of previously tried hyperparameter configurations and their corresponding performance metrics. Initially, $D$ is empty and is populated as the algorithm evaluates new configurations.

The algorithm runs in a loop, repeating its process until it meets a predefined stopping condition, such as reaching a set number of iterations or exceeding a time limit. In each iteration, with probability $\rho$, the algorithm selects a new hyperparameter configuration at random. This step ensures diversity in the search process and prevents the algorithm from becoming trapped in local optima. If a random configuration is not selected, the algorithm proceeds to model-based selection.

During model-based selection, the algorithm searches for the highest budget $b$, where the historical data $D$ contains at least $N_{min}+2$ observations. Here, $N_{min}$​ represents the minimum number of data points required to fit the model reliably. If no such budget exists, the algorithm defaults to random sampling.

After identifying a suitable budget $b$, the algorithm divides the corresponding data subset $D_{b}$​ into two groups. The top-performing configurations, constituting the top $q$ fraction based on performance, are placed into $D_{good}$​, and the rest are assigned to $D_{bad}$​. The parameter $q$ denotes the top percentile threshold and determines the proportion of configurations considered as good performers.

The algorithm then fits two KDEs: $l(x)$ using $D_{good}$​ and $g(x)$ using $D_{bad}$​. These KDEs model the probability distributions of the good and bad configurations, respectively. To encourage exploration around promising regions, the bandwidths of the KDEs are scaled by a factor $b_{w}$​.

The general formula for the univariate KDE is (1):

$\hat{f}(x)=\frac{1}{nh}\sum_{i=1}^{n} K\left( \frac{x-x_{i}}{h} \right)$ (28)

In this context, $\hat{f}(x)$ denotes the estimated probability density function at point $x$. The variable $n$ represents the number of data points. The parameter $h$ is the bandwidth, which controls the smoothness of the resulting density curve. The function K is the kernel, typically a symmetric and positive function that integrates into one, such as the Gaussian kernel. Each $x_{i}$ represents an individual data point.

BOHB utilizes a multivariate extension of KDE to process multiple hyperparameters simultaneously. This method estimates the joint probability distribution over the hyperparameter space, enabling the algorithm to capture relationships between different hyperparameters.

Next, the algorithm generates $N_{s}$ candidate hyperparameter configurations by sampling from the KDE $l(x)$. For each candidate $x_{candidate}$, the algorithm computes the ratio $\frac{l\left( x_{candidate} \right)}{g (x_{candidate})}$, which indicates how much more likely $x$ is to be a good configuration rather than a bad one. The candidate with the highest likelihood ratio is selected for the next configuration evaluation.

By repeating this process, BOHB efficiently explores the hyperparameter space, striking a balance between exploring new configurations and exploiting known good ones. This strategy enables the identification of high-performing hyperparameter settings while effectively managing computational resources.

| Algorithm 2: Pseudo-code for hyperparameter optimization using BOHB. |
| --- |
| Input: Random sampling probability $\rho,$top percentile threshold $q,$number of candidate samples $N_{s},$minimum number of data points to fit model $N_{min},$bandwidth scaling factor $b_{w}$  Initialize historical evaluation data $D \leftarrow\emptyset$  While stopping criterion not met do:  If $rand() < \rho$:  Sample a random configuration $x$  Else:  Select the largest budget $b$ such that $\mid D_{b}\mid\geq N_{min}+2$  If no such budget exists:  Sample a random configuration $x$  Else:  Partition $D_{b}$ into:  $D_{good}$: top $q\times\mid D_{b}\mid$ configurations based on performance  $D_{bad}$: remaining configurations  Fit two KDEs:  $l(x)$ using $D_{good}$  $g\left( x \right)$ using $D_{bad}$  Generate $N_{s}$​ candidate samples from $l(x)$ using bandwidths scaled by $b_{w}$  For each sample $x_{candidate}$:  Calculate the likelihood ratio $\frac{l(x_{candidate})}{g(x_{candidate})}$  Select the sample $x$ with the highest ratio  Evaluate configuration $x$ and update $D$ with the result |

**References**

1. Dogan V, Prestwich S, editors. BHO-MA: Bayesian Hyperparameter Optimization with Multi-objective Acquisition. International Conference on Optimization, Learning Algorithms and Applications; 2023: Springer.
